# Supplementary material for: Modulation of brain cation-Cl− cotransport via the SPAK kinase inhibitor ZT-1a
Source: Nat Commun. 2020 Jan 7;11:78. doi: 10.1038/s41467-019-13851-6 (PMC6946680; doi:10.1038/s41467-019-13851-6)
Supplement: Supplementary file 1 — Supplementary Information [file 41467_2019_13851_MOESM1_ESM.pdf]

Supplementary Information

Modulation of brain cation-Cl^- cotransport via the SPAK kinase inhibitor ZT-1a

Zhang *et al*.

MATERIALS AND METHODS

I. General Methods for Chemistry.

Reagents and solvents were obtained from commercial sources and used without further purification. Reactions were monitored by thin layer chromatography (TLC) on glass plates coated with silica gel with fluorescent indicator. Compounds were purified either by chromatography on silica gel or by preparative high performance liquid chromatography (HPLC). Silica gel chromatography was performed on the Teledyne ISCO CombiFlash system (RF200) eluting with petroleum ether/ethyl acetate (PE/EA) or dichloromethane/methanol (DCM/MeOH). Preparative HPLC was conducted on the Waters autopurification system consisting of a 2767 sample manager, a 2545 binary gradient module, a 2489 UV detector, and a 3100 mass detector. NMR spectra were recorded on a Bruker Ultrashield Plus-600 (600 MHz) spectrometer and chemical shifts are reported in δ (ppm). ^{1}H chemical shifts are reported as s (singlet), d (doublet), dd (doublet of doublet), t (triplet), q (quartet), m (multiplet), and brs (broad singlet) and are referenced to the residual solvent signal: DMSO-d_6 (2.50). ^{13}C spectra are referenced to the residual solvent signal: DMSO-d_6 (39.52). High Resolution Mass Spectra were obtained on a Thermo Fisher Scientific LTQ FTICR-MS. The purity of all tested compounds was >95% by HPLC.

Synthesis of 1a-1j.

Synthetic scheme of **1a**

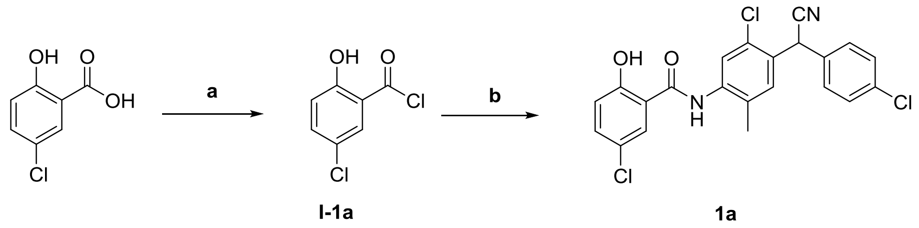

Reagents and conditions: a) $SOCl_2$, reflux; **b)** 2-(4-amino-2-chloro-5-methylphenyl)-2-(4-chlorophenyl)acetonitrile, Dioxane, 50 ℃,1 h

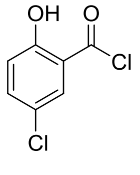

I-1a

5-chloro-2-hydroxybenzoyl chloride **(I-1a)** The mixture of 5-chloro-2-hydroxy-benzoic acid (103.5 mg, 0.6 mmol) in 1.5 mL of thionyl chloride was refluxed at 80°C for 2 h. The resulting solution was cooled to room temperature, and excess thionyl chloride was removed under vacuum to afford **I-1a** as gummy yellow solid, **I-1a** was directly used in the next step.

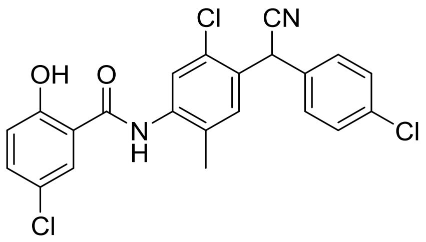

1a (ZT-1a)

***5-chloro-N-(5-chloro-4-((4-chlorophenyl)(cyano)methyl)-2-methylphenyl)-2-hydroxy-benzamide*(1a)**

To a stirred solution of 2-(4-amino-2-chloro-5-methylphenyl)-2-(4-chlorophenyl)acetonitrile (87.3 mg, 0.3 mmol) in 1,4-dioxane (4 mL) was added **I-1a** (85.9 mg, 0.45 mmol) at room temperature, and the resulting mixture was heated at 50°C for 1 h. Then the reaction was cooled to room temperature and extracted with ethyl acetate (20 mL). The organic extract was washed with brine (20 mL), dried over anhydrous $MgSO_4$, and concentrated in vacuum. The crude solid was purified by column chromatography on silica gel (dichloromethane /petroleum ether =3/1) to afford **1a** as white crystals, with a yield of 60.9 mg, 45%. ^{1}H NMR (600 MHz, DMSO-d_6) δ 12.20 (s, 1H), 10.48 (s, 1H), 8.29 (s, 1H), 7.96 (d, J = 2.8 Hz, 1H), 7.53 – 7.48 (m, 4H), 7.40 (d, J = 8.5 Hz, 2H), 7.07 (d, J = 8.8 Hz, 1H), 5.89 (s, 1H), 2.34 (s, 3H). ^{13}C NMR (150 MHz, DMSO-d_6) δ 163.6, 156.1, 138.4, 134.3, 133.8, 133.4, 131.9, 130.1, 130.0, 129.6, 129.4, 128.9, 123.9, 123.4, 120.2, 119.5, 38.5, 17.7. MS (ESI) *m/z*: 445[M+H]$^+$. HRMS (ESI) calculated for $C_{22}H_{14}Cl_3N_2O_2$ [M - H]$^-$, 443.0121; found, 443.0115.

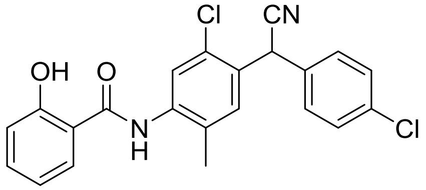

1b

N-(5-chloro-4-((4-chlorophenyl)(cyano)methyl)-2-methylphenyl)-2-hydroxy-benzamide Compound **1b** (42.7 mg, 34.6%) was prepared according to the methods described in the synthesis of **1a**. ^{1}H NMR (600 MHz, DMSO-d_6) δ 9.90 (s, 1H), 9.79 (s, 1H), 7.63 (s, 1H), 7.52 – 7.51 (m, 2H), 7.51 – 7.50 (m, 1H), 7.43 – 7.42 (m, 1H), 7.41 – 7.39 (m, 2H), 7.35 (dd, J = 3.7, 1.8 Hz, 1H), 7.33 (d, J = 7.8 Hz, 1H), 7.02 – 6.98 (m, 1H), 6.03 (s, 1H), 2.29 (s, 3H). ^{13}C NMR (150 MHz, DMSO-d_6) δ 166.1, 157.9, 138.6, 136.0, 134.3, 133.8, 133.4, 132.0, 130.3, 130.0, 130.0, 129.6, 129.5, 127.6, 119.5, 119.3, 118.7, 115.1, 38.6, 18.0. MS (ESI) *m/z*: 411[M+H]$^+$. HRMS (ESI) calculated for $C_{22}H_{17}Cl_2N_2O_2$ [M+H]$^+$, 411.0662; found, 411.0662.

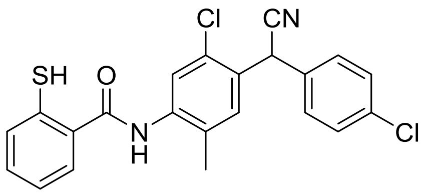

1c

***N*-(5-chloro-4-((4-chlorophenyl)(cyano)methyl)-2-methylphenyl)-2-mercapto-benzamide** Compound **1c** (20 mg, 31.2%) was prepared according to the methods described in the synthesis of **1a**. ^{1}H NMR (600 MHz, DMSO-d_6) δ 8.10 – 8.01 (m, 1H), 7.96 (dt, *J* = 7.8, 1.0 Hz, 1H), 7.77 (ddd, *J* = 8.4, 7.2, 1.3 Hz, 1H), 7.69 (s, 2H), 7.54 – 7.50 (m, 3H), 7.49 – 7.44 (m, 2H), 6.12 (s, 1H), 2.19 (s, 3H). ^{13}C NMR (150 MHz, DMSO-d_6) δ 163.6, 141.4, 137.4, 136.3, 133.7, 133.1, 133.0, 132.4, 132.1, 130.7, 129.7, 129.6, 129.1, 126.1, 125.8, 122.9, 122.0, 118.7, 38.1, 17.1. MS (ESI) *m/z*: 427 $[M+H]^+$. HRMS (ESI) calculated for $C_{22}H_{15}Cl_2N_2OS$ $[M - H]^-$, 425.0282; found, 425.0277.

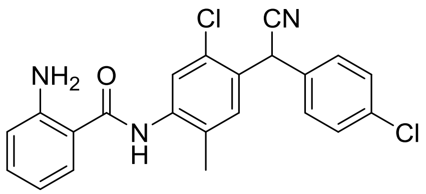

1d

2-amino-N-(5-chloro-4-((4-chlorophenyl)(cyano)methyl)-2-methylphenyl)-benzamide To a stirred solution of **1e** (41.0 mg, 0.1 mmol) in acetic acid (1mL) was added metallic iron powder (16.8 mg, 0.3 mmol) at room temperature. The reaction mixture was heated to 56℃ for 1.5 h and then cooled to room temperature. The solvent was removed under reduced pressure and extracted with ethyl acetate. The organic layer was washed with brine, dried over anhydrous $MgSO_4$, and concentrated under reduced pressure. The crude solid was purified by column chromatography on silica gel (dichloromethane /petroleum ether =3/1) to afford **1d** as white crystals, with a yield of 20.6 mg, 50.2%. ^{1}H NMR (600 MHz, DMSO-d_6) δ 9.73 (s, 1H), 7.69 (d, *J* = 7.1 Hz, 1H), 7.61 (s, 1H), 7.52 (s, 1H), 7.51 (d, *J* = 2.4 Hz, 2H), 7.41 (d, *J* = 8.5 Hz, 2H), 7.22 (dd, *J* = 11.2, 4.1 Hz, 1H), 6.77 (d, *J* = 8.3 Hz, 1H), 6.61 (t, *J* = 7.5 Hz, 1H), 6.03 (s, 1H), 2.28 (s, 3H).^{13}C NMR (150 MHz, DMSO-d_6) δ 168.3, 150.4, 138.8, 134.3, 133.8, 133.4, 132.9, 131.9, 130.0, 129.6, 129.5, 129.3, 127.6, 119.6, 117.1, 115.4, 114.7, 110.0, 38.5, 18.0. MS (ESI) *m/z*: 410 $[M+H]^+$. HRMS (ESI) calculated for $C_{22}H_{18}Cl_2N_3O$ $[M + H]^+$, 410.0821; found, 410.0821.

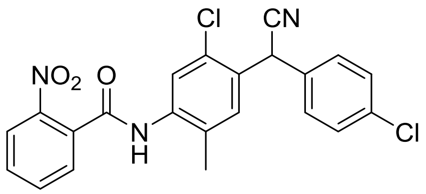

1e

N-(5-chloro-4-((4-chlorophenyl)(cyano)methyl)-2-methylphenyl)-2-nitrobenzamide Compound **1e** (78.5 mg, 59.4%) was prepared according to the methods described in the synthesis of **1a**. ^{1}H NMR (600 MHz, DMSO-d_6) δ 10.33 (s, 1H), 8.19 (d, *J* = 8.1 Hz, 1H), 7.91 (t, *J* = 7.4 Hz, 1H), 7.84 (d, *J* = 7.3 Hz, 1H), 7.78 (s, 1H), 7.74 (s, 1H), 7.52 (d, *J* = 1.2 Hz, 2H), 7.50 (s, 1H), 7.41 (d, *J* = 8.4 Hz, 2H), 6.05 (s, 1H), 2.30 (s, 3H). ^{13}C NMR (150 MHz, DMSO-d_6) δ 165.3, 146.8, 137.7, 134.7, 134.2, 133.4, 132.8, 132.7, 132.2, 131.5, 130.3, 130.0, 129.9, 129.7, 129.6, 126.6, 124.8, 119.5, 38.6, 17.8. MS (ESI) *m/z*: 440[M+H]$^+$. HRMS (ESI) calculated for $C_{22}H_{14}Cl_2N_3O_3$ [M - H]$^-$, 438.0412; found, 438.0407.

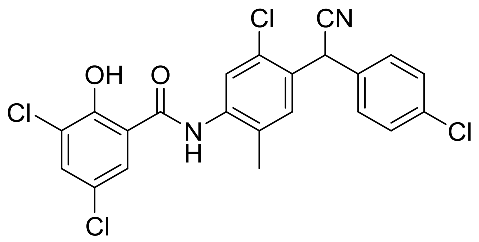

1f

3,5-dichloro-N-(5-chloro-4-((4-chlorophenyl)(cyano)methyl)-2-methylphenyl)-2-hydroxy-benzamide Compound **1f** (78.5 mg, 59.4%) was prepared according to the methods described in the synthesis of **1a**. ^{1}H NMR (600 MHz, DMSO-d_6) δ 10.63 (s, 1H), 8.05 (d, *J* = 2.5 Hz, 1H), 7.84 (d, *J* = 2.5 Hz, 1H), 7.76 (d, *J* = 2.7 Hz, 1H), 7.75 – 7.73 (m, 1H), 7.55 (s, 1H), 7.52 – 7.48 (m, 2H), 7.43 – 7.39 (m, 2H), 6.04 (s, 1H), 2.29 (s, 3H). ^{13}C NMR (150 MHz, DMSO-d_6) δ 166.0, 136.7, 133.7, 133.1, 133.0, 133.0, 131.6, 130.5, 129.6, 129.3, 129.2, 128.3, 126.9, 126.8, 122.9, 122.5, 119.0, 118.8, 38.1, 17.3. MS (ESI) *m/z*: 479 [M+H]$^+$. HRMS (ESI) calculated for $C_{22}H_{13}Cl_4N_2O_2$ [M - H]$^-$, 476.9731; found, 476.9726.

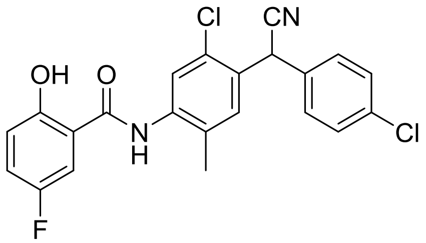

1g

N-(5-chloro-4-((4-chlorophenyl)(cyano)methyl)-2-methylphenyl)-5-fluoro-2-hydroxy-benzamide Compound **1g** (78.5 mg, 59.4%) was prepared according to the methods described in the synthesis of **1a**. ^{1}H NMR (600 MHz, DMSO-d_6) δ 11.97 (s, 1H), 10.55 (s, 1H), 8.33 (s, 1H), 7.73 (dd, *J* = 9.6, 3.3 Hz, 1H), 7.53 – 7.50 (m, 2H), 7.50 – 7.48 (m, 1H), 7.43 – 7.38 (m, 2H), 7.33 (ddd, *J* = 8.9, 7.8, 3.3 Hz,

1H), 6.01 (s, 1H), 2.34 (s, 3H). MS (ESI) *m/z*: 429$[M+H]^+$. HRMS (ESI) calculated for $C_{22}H_{14}Cl_2FN_2O_2$ $[M - H]^-$, 427.0416; found, 427.0411.

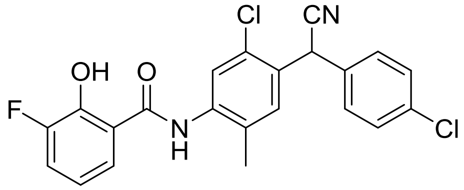

1h

***N*-(5-chloro-4-((4-chlorophenyl)(cyano)methyl)-2-methylphenyl)-3-fluoro-2-hydroxy-benzamide**

Compound **1h** (78.5 mg, 59.4%) was prepared according to the methods described in the synthesis of **1a**. ^{1}H NMR (600 MHz, DMSO-d_6) δ 12.24 (s, 1H), 10.61 (s, 1H), 8.11 (s, 1H), 7.85 (d, *J* = 8.0 Hz, 1H), 7.53 (s, 1H), 7.52 – 7.49 (m, 2H), 7.46 (ddd, *J* = 10.8, 8.1, 1.5 Hz, 1H), 7.42 – 7.39 (m, 2H), 6.99 (td, *J* = 8.1, 4.8 Hz, 1H), 6.03 (s, 1H), 2.33 (s, 3H). MS (ESI) *m/z*: 429$[M+H]^+$. HRMS (ESI) calculated for $C_{22}H_{14}C_{l2}FN_2O_2$ $[M - H]^-$, 427.0416; found, 427.0411.

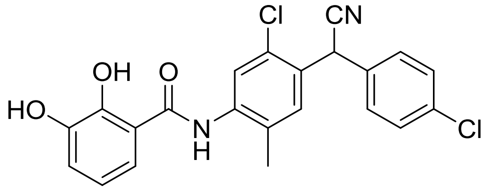

1i

***N*-(5-chloro-4-((4-chlorophenyl)(cyano)methyl)-2-methylphenyl)-2,3-dihydroxy-benzamide**

Compound **1i** (78.5 mg, 59.4%) was prepared according to the methods described in the synthesis of **1a**. ^{1}H NMR (600 MHz, DMSO-d_6) δ 11.26 (s, 1H), 10.56 (s, 1H), 9.77 (s, 1H), 8.19 (s,1H), 7.50 (dd, *J* = 6.7, 4.7 Hz, 3H), 7.46 (dd, *J* = 8.1, 1.5 Hz, 1H), 7.41 – 7.37 (m, 2H), 7.01 (dd, *J* = 7.8, 1.6 Hz, 1H), 6.86 – 6.77 (m, 1H), 6.02 (s, 1H), 2.31 (s, 3H). ^{13}C NMR (150 MHz, DMSO-d_6) δ 166.0, 147.0, 146.6, 138.5, 134.3, 133.4, 131.9, 130.1, 130.0, 130.0 129.6, 128.9, 124.1, 120.1, 119.6, 119.6, 119.5, 118.5, 38.5, 17.8. MS (ESI) *m/z*: 427$[M+H]^+$. HRMS (ESI) calculated for $C_{22}H_{15}Cl_2N_2O_3$ $[M - H]^-$, 425.0460; found, 425.0454.

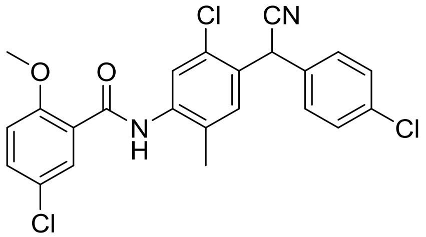

1j

5-chloro-N-(5-chloro-4-((4-chlorophenyl)(cyano)methyl)-2-methylphenyl)-2-methoxy-benzamide

Compound **1j** (78.5 mg, 59.4%) was prepared according to the methods described in the synthesis of **1a**. ^{1}H NMR (600 MHz, DMSO-d_6) δ 10.01 (s, 1H), 8.16 (s, 1H), 7.85 (d, *J* = 2.8 Hz, 1H), 7.63 (dd, *J* = 8.9, 2.8 Hz, 1H), 7.52 (s, 1H), 7.52 – 7.51 (m, 1H), 7.51 – 7.49 (m, 1H), 7.40 (d, *J* = 8.5 Hz, 1H), 7.31 (d, *J* = 8.9 Hz, 1H), 6.02 (s, 1H), 4.00 (s, 3H), 2.35 (s, 3H). ^{13}C NMR (150 MHz, DMSO-d_6) δ 162.8, 156.3, 138.3, 134.3, 133.4, 133.1, 131.9, 130.4, 130.0, 130.0, 129.9, 129.6, 129.1, 125.3, 124.5, 123.9, 119.6, 115.1, 57.3, 38.5, 17.6. MS (ESI) *m/z*: 459 $[M+H]^+$. HRMS (ESI) calculated for $C_{22}H_{15}Cl_2N_2O_3$ $[M - H]^-$, 457.0277; found, 457.0272.

II. Experimental procedures

1. Reagents and General methods

Tissue culture reagents were from Life Technologies. P81 phosphocellulose paper was from Whatman and [γ-^{32}P]-ATP was from Perkin Elmer. CATCHtide was synthesised by Pepceuticals. Protein G sepharose was from Amersham. DNA constructs used for transfection were purified from *Escherichia coli* DH5α using Qiagen or Invitrogen plasmid Maxi kits according to the manufacturer's protocol. All DNA constructs were verified by DNA sequencing.

2. Plasmids, protein expression and purification

DNA clones were from the Division of Signal Transduction Therapy (University of Dundee). The SPAK proteins were expressed in *E. Coli* and purified as described previously [1].

3. SPAK kinase assays and IC_{50} determination

SPAK kinase assays employed CATCHtide (cation chloride co-transporter peptide substrate). Peptide kinase assays, performed in triplicate, were set up in a total volume of 50 µl containing 0.5 µg GST–[T233E]SPAK kinase (~5 nM at ~10% purity) in 50 mM Tris/HCl, pH 7.5, 0.1 mM EGTA, 10 mM $MgCl_2$, 300 µM CATCHtide (RRHYYYDTHTNTYYLRTFGHNTRR), 0.1 µM [γ-32P]ATP (~500 cpm/pmol) and the indicated concentrations of inhibitor dissolved in DMSO. After incubation for 30 min at 30°C, reactions were terminated by spotting 40 µl of the reaction mix onto P81 phosphocellulose paper and immersion in 50 mM phosphoric acid. The papers were washed extensively and incorporation of [γ-^{32}P]ATP into CATCHtide was quantified by Cerenkov counting. IC_{50} values were calculated with GraphPad Prism using non-linear regression analysis.

4. Fluorescence polarization

Fluorescence polarization measurements were performed at 25°C with purified SPAK proteins in 50 mM Tris/HCl, pH 7.5, 150 mM NaCl and 2 mM DTT. The concentration of SPAK proteins was determined by measuring their absorbance at 280 nm and calculated using the molar absorption coefficient determined by the ProtParam Online tool[2]. All peptides (SEEGKPQLVG**RFQV**TSSK [EP4543] and SEEGKPQLVG**AFQV**TSSK [EP4544) contained an N-terminal linker required for conjugation to the Lumio Green fluorophore (CCPGCCGGGG) and were initially resuspended in 50 mM ammonium bicarbonate, pH 8. Peptide labelling was achieved by incubating 10 nM of each peptide in a 0.5 ml reaction mixture of 20 µM Lumio Green in 25 mM Tris/HCl, pH 7.5, 200 mM NaCl and 5 mM 2-mercaptoethanol. Reactions were left to proceed in the dark for 2 h. The peptides were dialyzed

for 4 h into 25 mM Tris/HCl, pH 7.5, 200 mM NaCl and 5 mM 2-mercaptoethanol using a Micro DispoDIALYZER with a 100 Da molecular-mass cut-off (Harvard Apparatus), and then for another 12 h with changed buffer. For fluorescence polarization, mixtures were set up containing the indicated concentration of protein and 10 nM Lumio-Green-labelled peptide in a final volume of 30 µl. All individual bindings were performed in duplicate with at least 12 data points per curve. Fluorescence polarization was measured using a BMG PheraStar plate reader, with an excitation wavelength of 485 nm and an emission wavelength of 538 nm, and measurements were corrected to the fluorescent probe alone. Data analysis and graphing were then performed in GraphPad Prism7; a one-site specific binding model was assumed (Y=Bmax*X/[Kd+X]) and the fitted dissociation constant computed. All experimental binding studies were repeated at least twice.

5. Pharmacodynamic (PD) study

Male C57BL/6j wild-type mice (6 weeks old, Charles River Laboratories Edinburgh UK) were treated with ZT-1a (in DMSO, Sigma) by subcutaneous injection at doses of 0, 3, 10, 30, 50 and 100 mg/kg. 50 mg/kg Closantel was administered as a comparative control. Vehicle control mice were treated with an equal volume of DMSO solution. One hour after administration, mice were sacrificed by cervical dislocation, and kidney and brain tissues were rapidly dissected and snap-frozen in liquid nitrogen. Age-matched wild-type littermates and $SPAK^{502A/502A}$ knock-in mice were also used as comparative controls[1]. The $SPAK^{502A/502A}$ knock-in mouse was established and maintained under specific pathogen-free conditions at the University of Dundee (UK) as described in our recent study[1]. Animal experiments and breeding were approved by the University of Dundee ethical committee and performed under a U.K. Home Office project license, in accordance with the Animals (Scientific Procedures) Act 1986, the Policy on the Care, Welfare and Treatment of Animals, and regulations set by the University of Dundee and the U.K. Home Office.

6. ZT-1a pharmacokinetic (PK) properties in sham control and ischemic stroke mice

ZT-1a content in brain homogenates and plasma was assayed at the University of Pittsburgh Small Molecule Biomarker Core, using liquid chromatography-tandem mass spectrometry (LC-MS/MS). C57BL/6j mice (male, 11 weeks old) at 3 hr post Sham surgery or tMCAO were intraperitoneally injected with 5 mg/kg ZT-1a. At 2 hr post injection, blood was collected into heparin-treated EP tubes by a cardiac puncture. After a brief cardiac perfusion with ice-cold normal saline, brain tissues (contralateral and ipsilateral hemispheres) were collected. Plasma samples were prepared by centrifugation of whole blood for 10 min at 1,500g using a refrigerated centrifuge. Brain tissues and

plasma samples were diluted, and protein was precipitated with acetonitrile prior to detection of ZT-1a with a triple quad mass spectrometer. Glyburide was used as the internal standard. Samples were then injected by autosampler and ZT-1a was eluted from a Waters Acquity UPLC BEH C18, 1.7 um, 2.1x100 mm reversed-phase column with a water (with 0.1% formic acid) and acetonitrile (with 0.1% formic acid) gradient. Detection and quantitation of ZT-1a were achieved in the positive mode with a Thermo Fisher TSQ Quantum Ultra mass spectrometer interfaced via an electrospray ionization (ESI) probe with the Water UPLC Acquity solvent delivery system.

7. Human fibroblasts

Primary fibroblast cell culture was established as described previously[3]. Cells ($2x10^5$ cells per coverslip) were cultured on glass coverslips coated with poly-D-lysine in Dulbecco's Modified Eagle Medium (DMEM) supplemented with 10% fetal bovine serum (FBS) and 0.1 mg/mL penicillin/streptomycin Cultures were incubated at 37^oC in an incubator with 5% CO_2 and atmospheric air. Passages 9-21 were used in the study.

8. Cell volume measurements

Cell volume change was determined using the dye Calcein AM as a marker of intracellular water volume, as described previously[4]. The cells plated on the coverslip were incubated with 0.5 µM calcein-AM for 30 min at 37°C in the dark. The coverslip was then mounted in a heated (37°C) imaging/perfusion chamber (Warner Instruments, Hamden, CT) on a Nikon Ti Eclipse inverted epifluorescence microscope equipped with perfect focus, a 40X Super Fluor oil immersion objective lens, and a Princeton Instruments MicroMax CCD camera. Calcein fluorescence was monitored using a FITC filter set (excitation 480 nm, emission 535 nm, Chroma Technology, Rockingham, VT). Images were collected every 60 secs with MetaFluor image-acquisition software (Molecular Devices, Sunnyvale, CA) and regions of interest (~20-25 cells) were selected. Baseline drift resulting from photobleaching or dye leakage was corrected as described before[5]. The fluorescence change was plotted as a function of the reciprocal of the relative osmotic pressure and the resulting calibration curve applied to all subsequent experiments as described[5]. HEPES-buffered isotonic solution (contained-confirmed at 310 mOsm, Advanced Instruments, Norwood, MA) contained (in mM, pH 7.4): 100 NaCl, 5.4 KCl, 1.3 $CaCl_2$, 0.8 $MgSO_4$, 20 HEPES, 5.5 glucose, 0.4 $NaHCO_3$, and 70 sucrose. Anisosmotic solutions (250, 370, 400, 515 mOsm) were prepared by removal or addition of sucrose to the above solution.

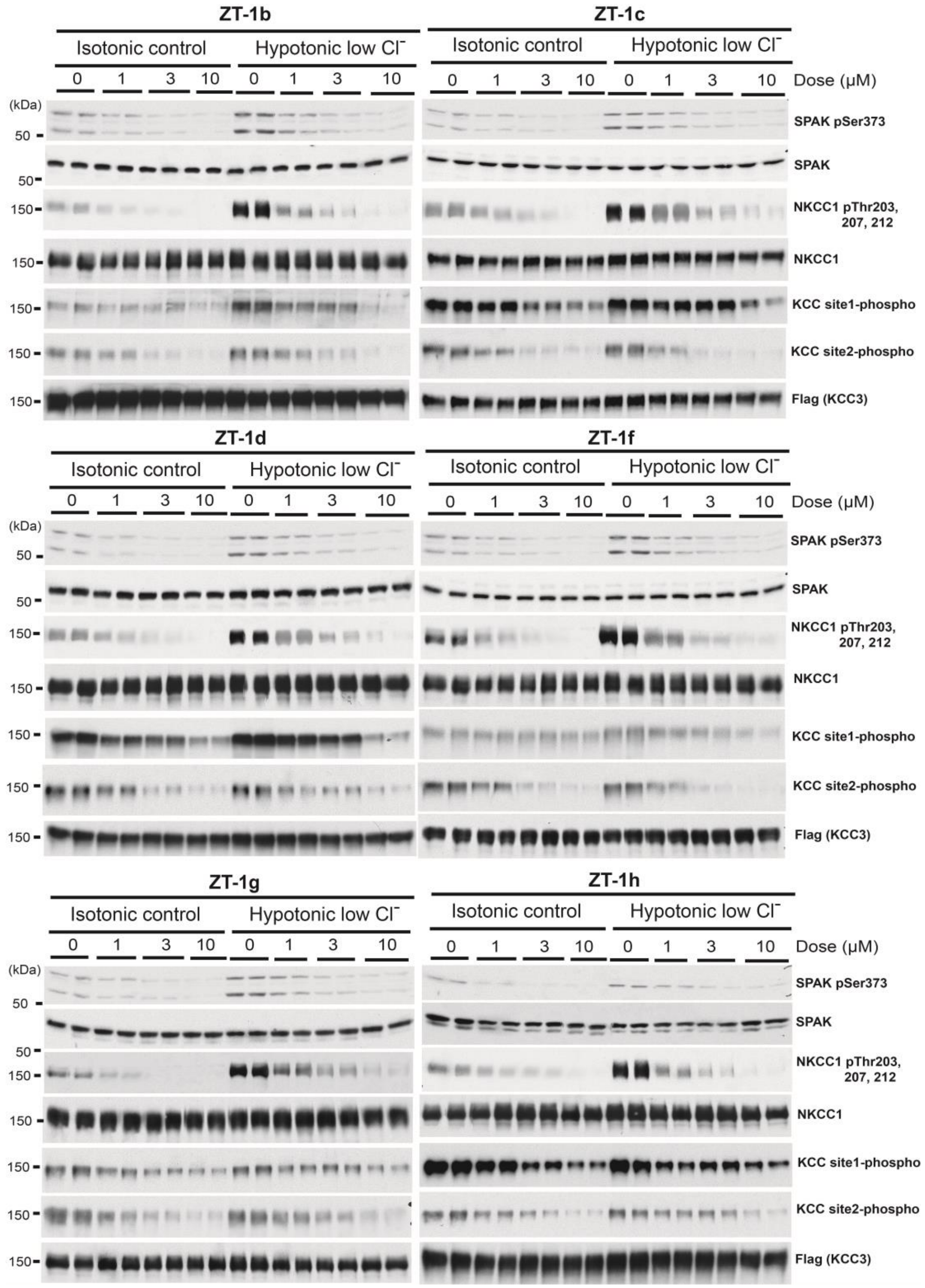

Supplemental Fig. 1 Concentration-response experiments testing effects of Closantel analogues on phosphorylation of KCC3 Thr991/Thr1048. HEK293 cells were transfected with cDNA encoding wild type N-terminally FLAG-tagged KCC3. 36 h post-transfection, cells were exposed 30 min to either control isotonic conditions or hypotonic low Cl^- conditions, then treated in the same conditions with the specified inhibitors (1b, 1c, 1f, 1d, 1h and 1g) at the indicated concentrations for an additional 30 min. Lysates were subjected to SDS-PAGE and Western blotting with the indicated antibodies. See **Supplementary Fig. 2** for immunoblot quantitation.

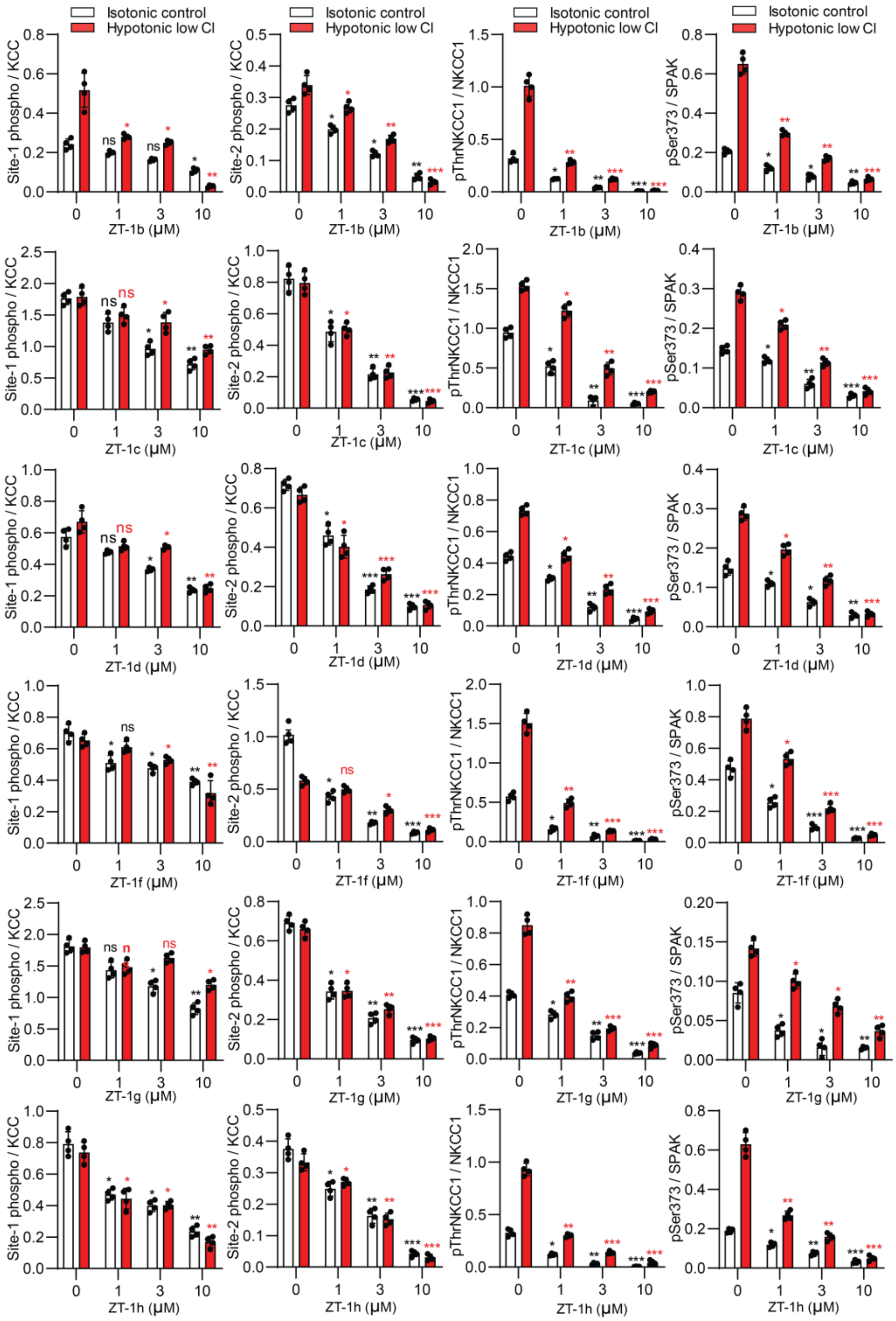

Supplemental Fig. 2 Concentration-response experiments testing effects of Closantel analogues on phosphorylation of KCC3 Thr991/Thr1048.

HEK293 cells were transfected with cDNA encoding wild type N-terminally FLAG-tagged KCC3. 36 h post-transfection, cells were exposed 30 min to either control isotonic conditions or hypotonic low Cl- conditions, then treated in the same conditions with the specified inhibitors (1b, 1c, 1f, 1d, 1h and 1g) at the indicated concentrations for an additional 30 min. Lysates were subjected to SDS-PAGE and Western blotting with the indicated antibodies. See **Supplementary Fig. 1** for Western blots. ***, $p < 0.001$; **, $p < 0.01$; *, $p < 0.05$ versus DMSO control, one-way ANOVA.

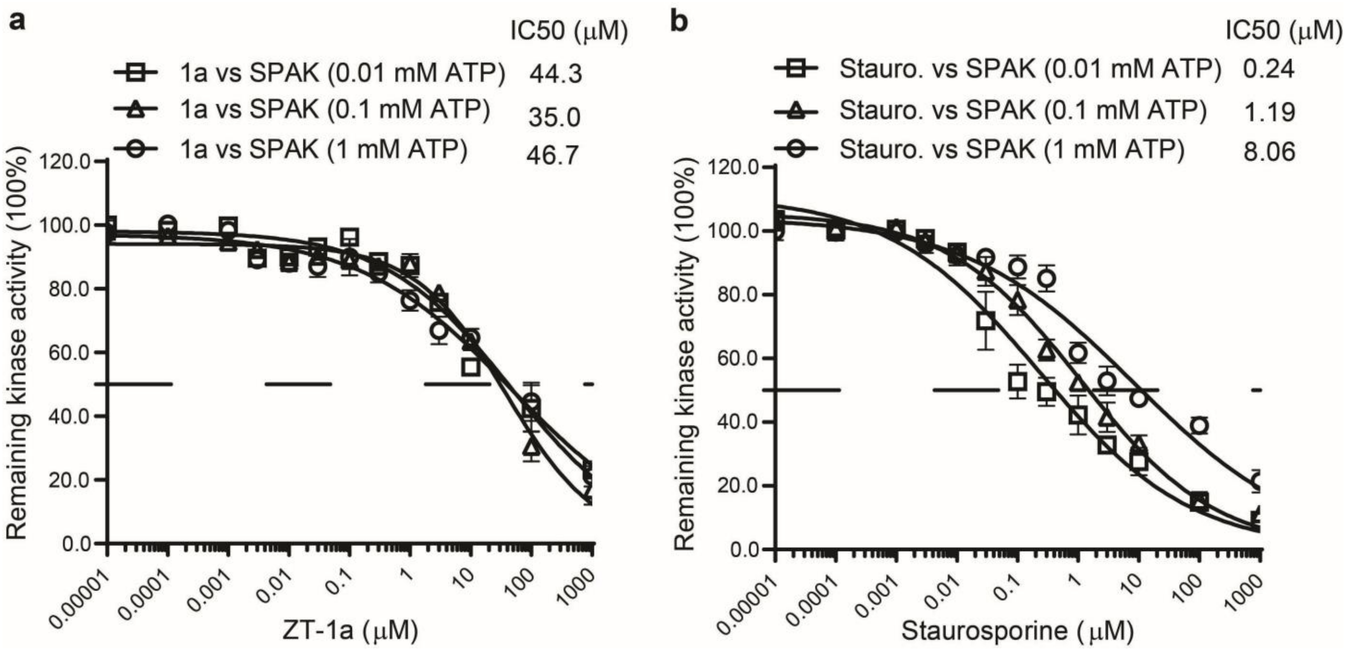

Supplemental Fig. 3 Effects of increasing concentrations of ZT-1a and staurosporine on SPAK activity at ATP concentrations between 0.01 and 1 mM. ZT-1a and staurosporine were added at concentrations between 0.00001 to 1 mM. Data points are the means of three determinations, with error bars ± SEM. Staurosporine IC_{50} values increased as [ATP] increased, whereas ZT-1a IC_{50} results were unaffected by changing [ATP].

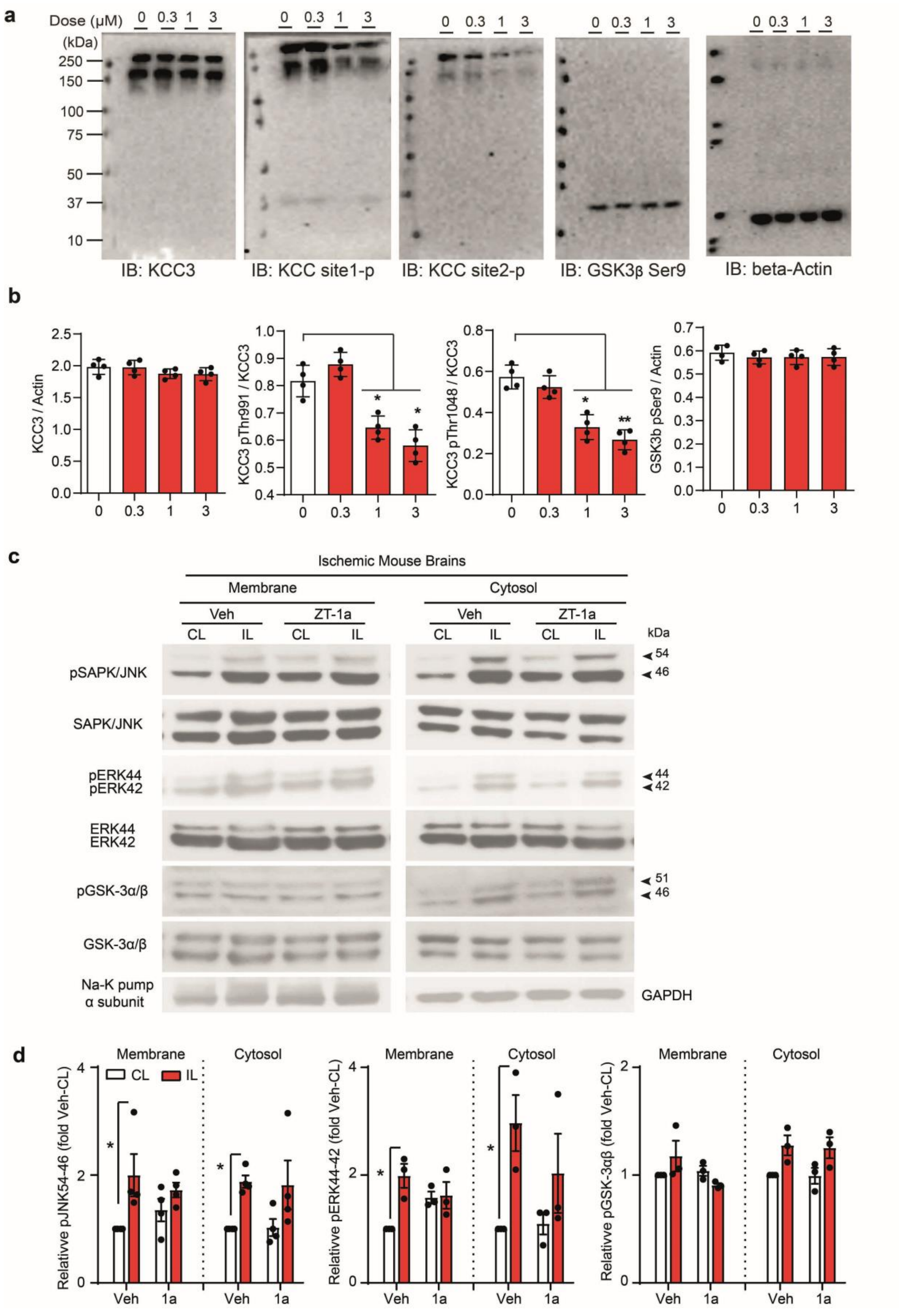

Supplemental Fig. 4 ZT-1a treatment does not affect Akt-mediated phosphorylation at Ser9 of GSK-3β. a HEK293 cells were transfected with cDNA encoding wild type N-terminally FLAG-tagged

KCC3. 36 h post-transfection, cells were treated with ZT-1a at the indicated concentrations for 30 min. Lysates were subjected to SDS-PAGE and immunoblot with antibodies specific for total KCC3, KCC3 pThr991, KCC3 pThr1048, SGK3β pSer9 and Actin. **b** Densitometric analyses of immunoblots. Data are means ± SEM, n = 3. * $p < 0.05$; ** $p < 0.01$ vs. absence of ZT-1a. **c** Representative immunoblots (IB) of phospho-GSK-3α/β (pGSK-3α/β), phospho-JNK (pJNK) and phospho-ERK1/2 (pERK1/2) in mouse brains at 24 h post-reperfusion after ischemic stroke. Cytosol and crude membrane protein fractions were prepared from contralateral (CL) and ipsilateral (IL) cerebral hemispheres. Vehicle (DMSO, 2 ml/kg) or ZT-1a (5.0 mg/kg) were administered via intra-peritoneal injection (i.p.) with an initial half dose at 3 hr and the second half dose at 8 hr reperfusion via. Expressions of Na^+-K^+ ATPase α-subunit and GAPDH were probed as loading control for membrane and cytosol protein samples, respectively. **d** Densitometric analyses of immunoblots. Data are means ± SEM, n = 3. * $p < 0.05$ vs. absence of ZT-1a.

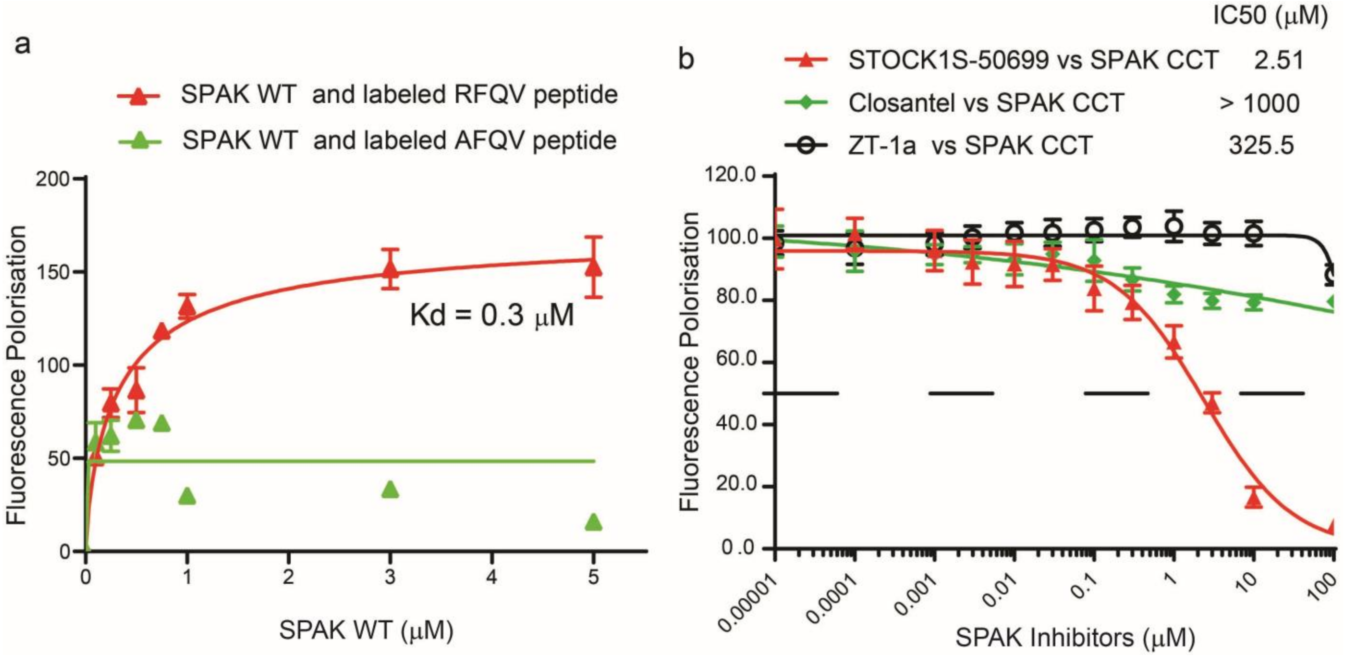

Supplemental Fig. 5 Evidence that SPAK associates with WNK1 in a manner disrupted by ZT-1a. **a** Analysis of SPAK–WNK interaction by fluorescence polarization. Purified human SPAK 452-547(C-term) and human SPAK 452-547 with L491A (equivalent to mouse SPAK L502) were appropriately diluted and mixed 1:1 (vol:vol) with 20 nM Lumino-Green-labelled WNK peptide (RFQV or AFQV) to the indicated concentration (with 10 nM final Lumino-Green WNK peptide concentration in each sample). Fluorescent polarization measurements were made, and binding curves, assuming one-site-specific binding, were generated with Prism6 using milli-polarization (mP) units. **b** Under the assay conditions used in (A), concentration-dependent decreases in fluorescence polarization were determined for STOCK1S-50699 (IC_{50} 2.51 μM), Closantel (IC_{50} >1000 μM) and ZT-1a (IC_{50} 325.5 μM).

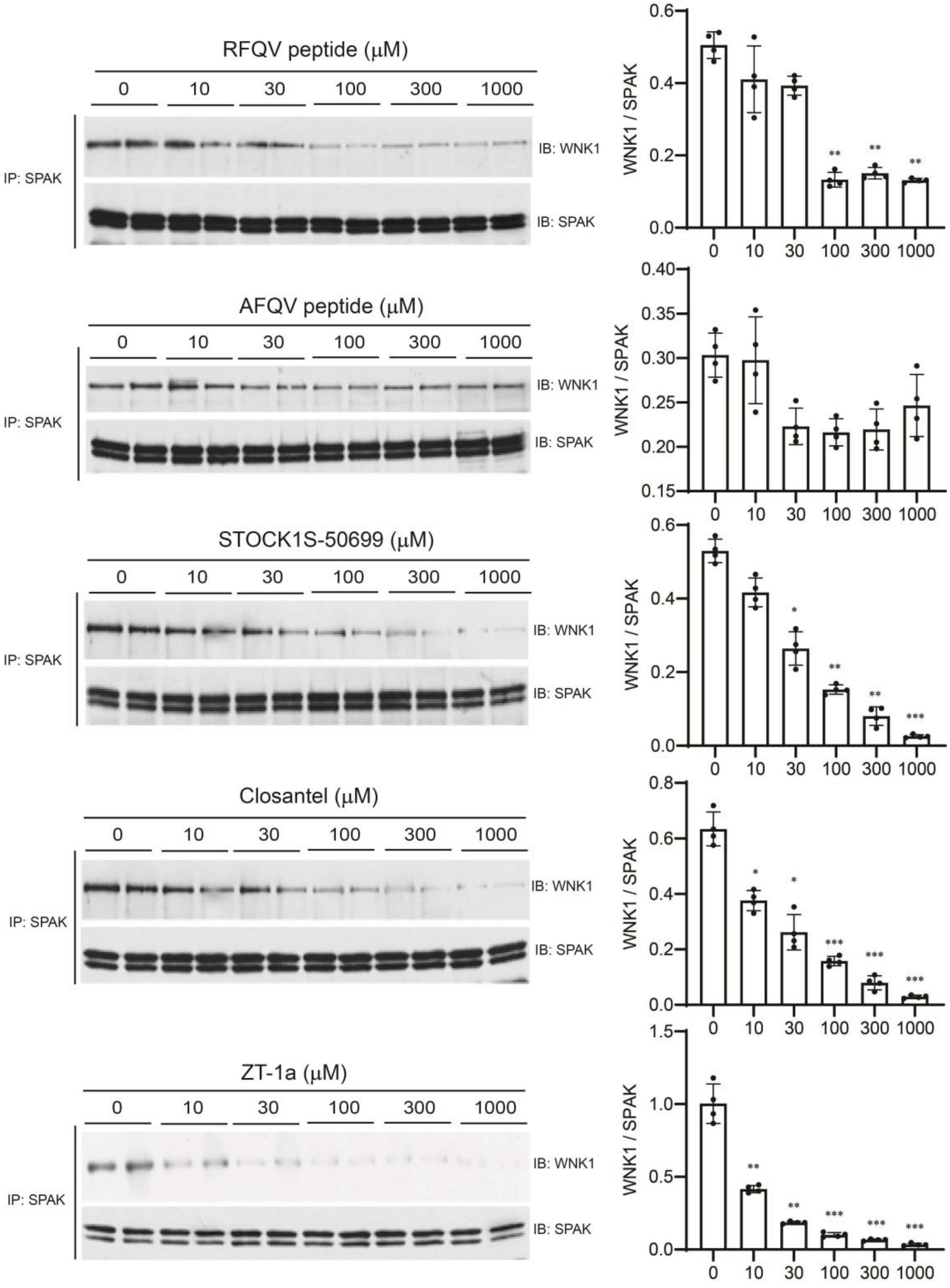

Supplemental Fig. 6 Evidence that SPAK associates with WNK1, and interaction is disrupted by ZT-1a and its analogs. Non-transfected HEK293 cell lysates were incubated with the RFQV peptide (SEEGKPQLVGRFQVTSSK), AFQV peptide (SEEGKPQLVGAFQVTSSK), STOCK1S-50699, Closantel or ZT-1a for 30 min on ice and subjected to SPAK antibody immunoprecipitation. Immunoprecipitates were subjected to immunoblot probed with antibody to total WNK1 and antibody to total SPAK. Left panels: representative immunoblots. Right panels, summarized data quantitated by densitometry (n=3, means ± SEM; **, $p < 0.01$)

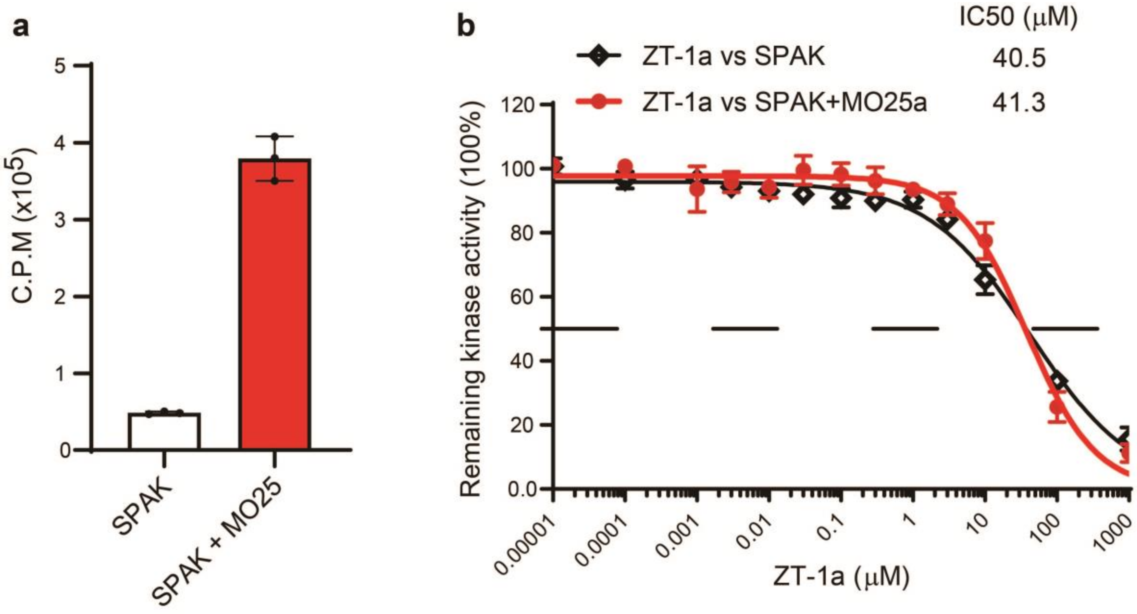

Supplemental Fig. 7 Effects of increasing concentrations of ZT-1a on SPAK activity in the absence or presence of wild-type MO25α. **a** Activation of SPAK kinase by MO25α. SPAK was assayed in the absence or presence of five-fold molar excess of wild-type MO25α. **b** Kinetic data determined for ZT-1a in the absence or presence of five-fold molar excess of wild-type MO25α. Data are means of three determinations ± SEM. IC_{50} results are 40.5 and 41.3 μM for ZT-1a in the respective absence or presence of wild-type MO25α.

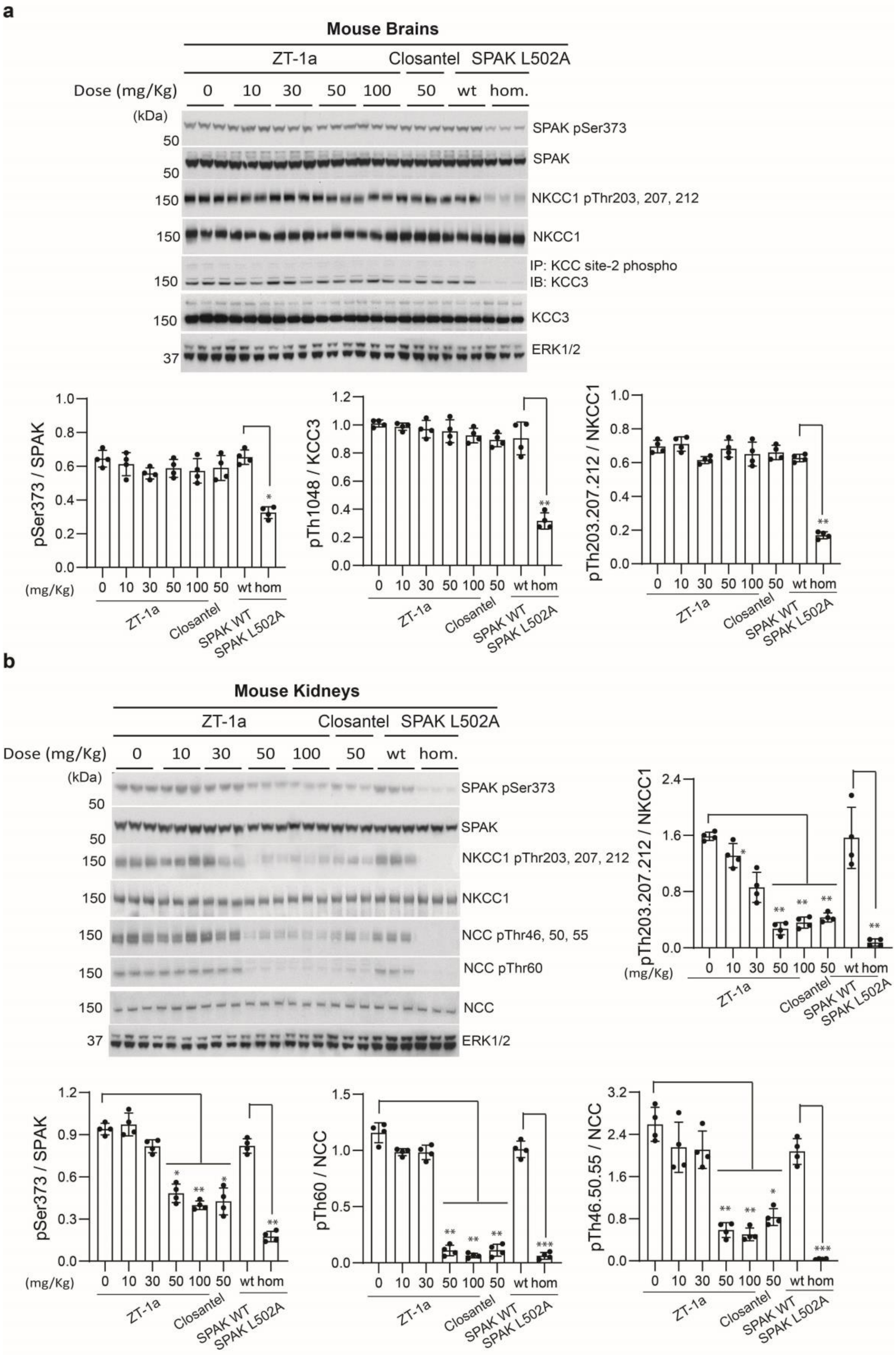

Supplemental Fig. 8 Pharmacodynamic analysis of ZT-1a and Closantel in brain and kidney tissues of naïve mice. Drugs were administered in naïve mice via intraperitoneal (i.p.) administration at

the indicated doses. Brain and kidney tissues were collected at 60 min post injection, and proteins subjected to immunoblot were probed with antibodies specific for phosphorylated forms of SPAK, NKCC1 and NCC. Age-matched wild-type and SPAK-L502A knock-in mice were used as controls. Quantitative densitometric data are means ± SEM for 3 experiments; *, $p<0.05$; **, $p<0.01$.

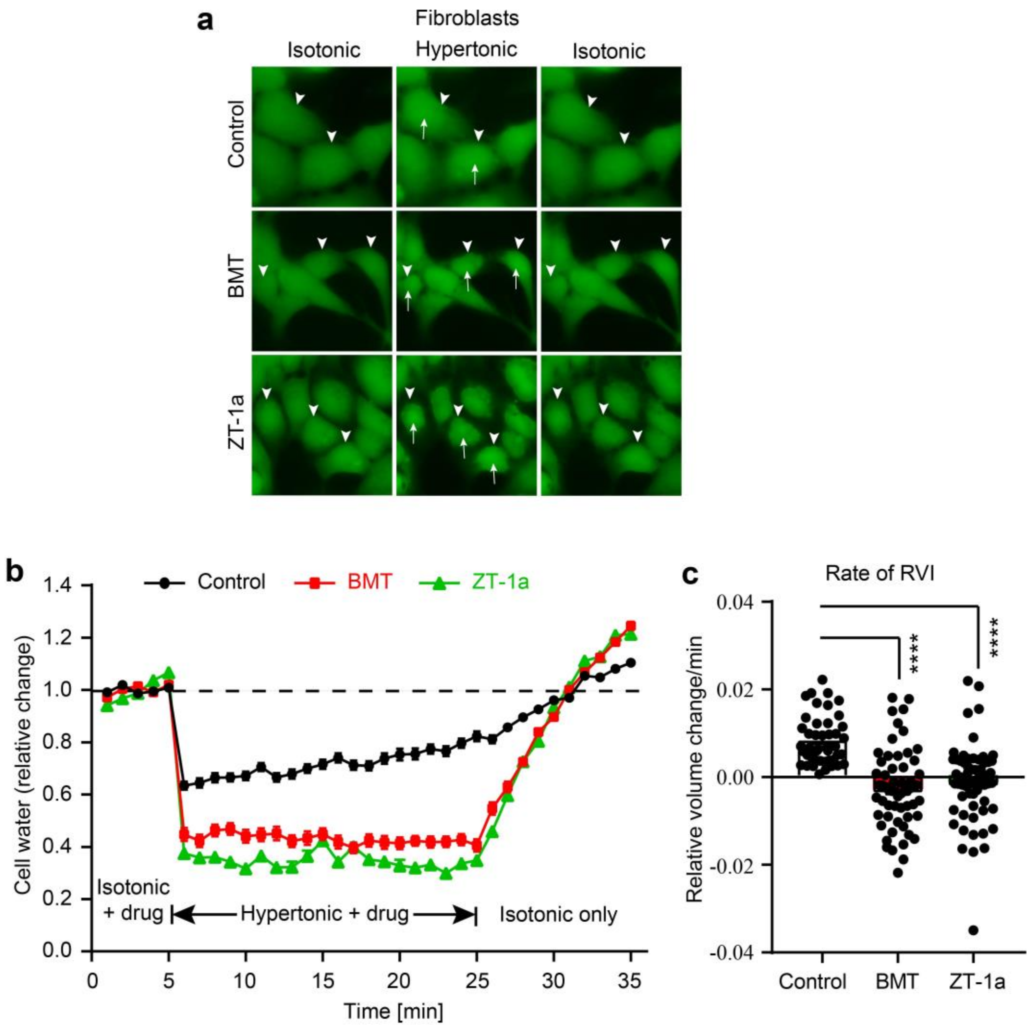

Supplementary Fig. 9 Effects of SPAK inhibitor ZT-1a on cell volume changes in human fibroblast cells.

a Representative images of human fibroblasts cells loaded with Calcein AM dye. Arrow-heads mark cells of interest; Arrows illustrate changes of fluorescence intensity in response to hyperosmotic stress. Cells were exposed to isotonic HEPES-MEM (310 mOsm, 5 min, +/- drug), hypertonic HEPES-MEM (515 mOsm, 20 min, +/- drug), and isotonic HEPES-MEM (10 min, no drug) in the absence or presence of SPAK inhibitor ZT-1a (3 μM), or the potent NKCC1 inhibitor bumetanide (BMT, 10 μM). **b** Relative cell water content changes as function of time during indicated bath conditions. **c** Summary data of rate of regulatory volume increase (RVI). The rate was calculated by determining the slope between minutes 8-25. Data are means ± SEM, n = 44 cells for control, n = 57 cells for BMT, n = 62 cells for ZT-1a collected from 3 independent experiments. **** $p < 0.01$ vs control.

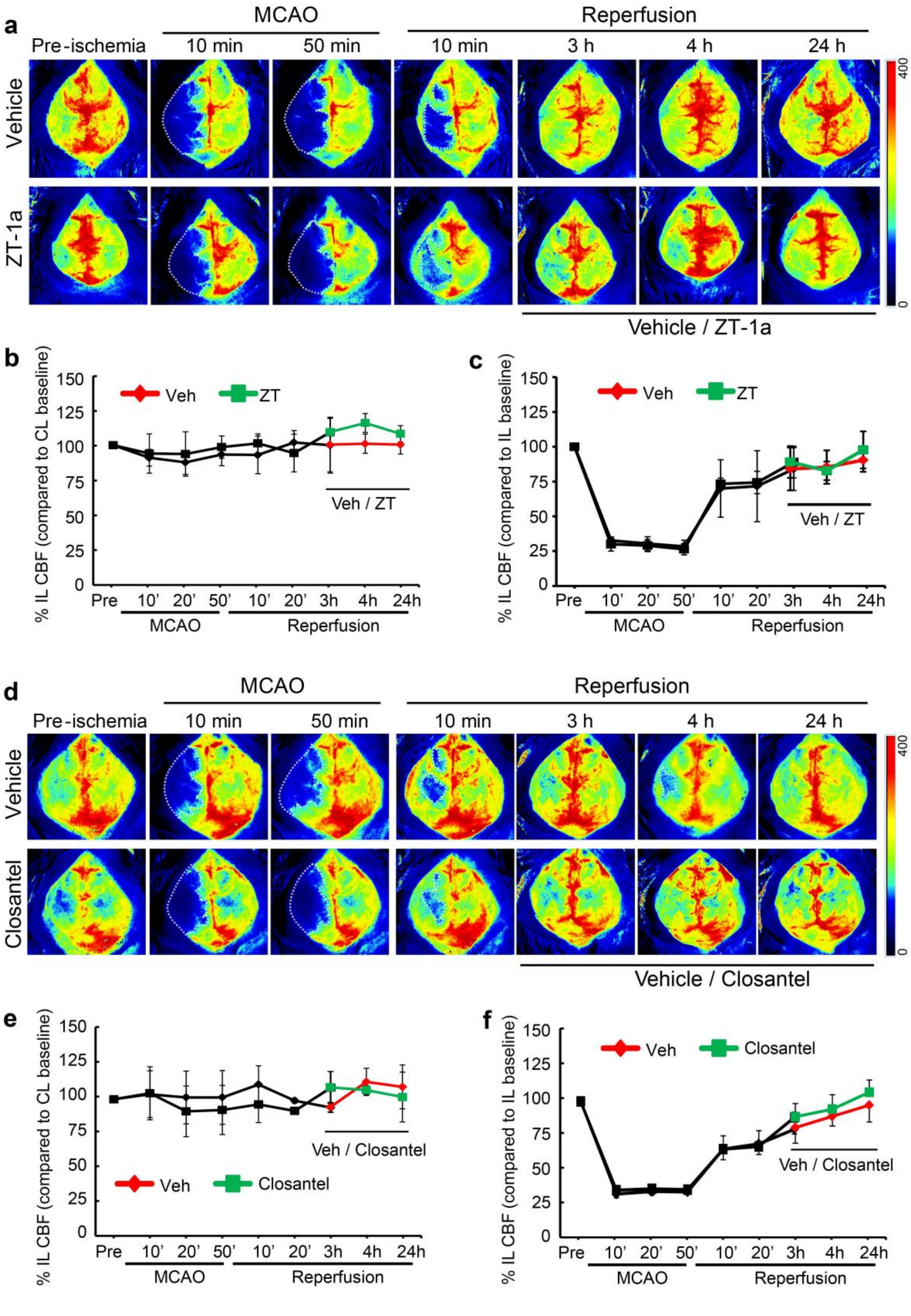

Supplemental Fig. 10 Post-stroke administration of ZT-1a or Closantel does not affect regional cerebral blood flow (rCBF) in ischemic mice. a and d Representative two-dimensional laser speckle contrast images of rCBF. Vehicle (DMSO), ZT-1a (A, 2.5 mg/kg), or Closantel (D, 1.25 mg/kg, i.p.) was administered i.p. at 3 hr and again at 8 hr post-reperfusion after MCAO. Pseudocolor scale bar indicates relative signal intensity. **b and e** No changes of rCBF were detected in the non-ischemic

contralateral (CL) hemispheres of vehicle-control or ZT-1a-treated (**b**) or Closantel-treated mice (**e**). Data are mean ± SD, n = 3. **c and f** Similar reductions and recoveries of rCBF in the ischemic ipsilateral hemispheres (IL) were detected in the ZT-1a-treated (**c**) or Closantel-treated ischemic mice (**f**), and neither differed from vehicle-injected mice. Data are mean ± SEM, n = 3.

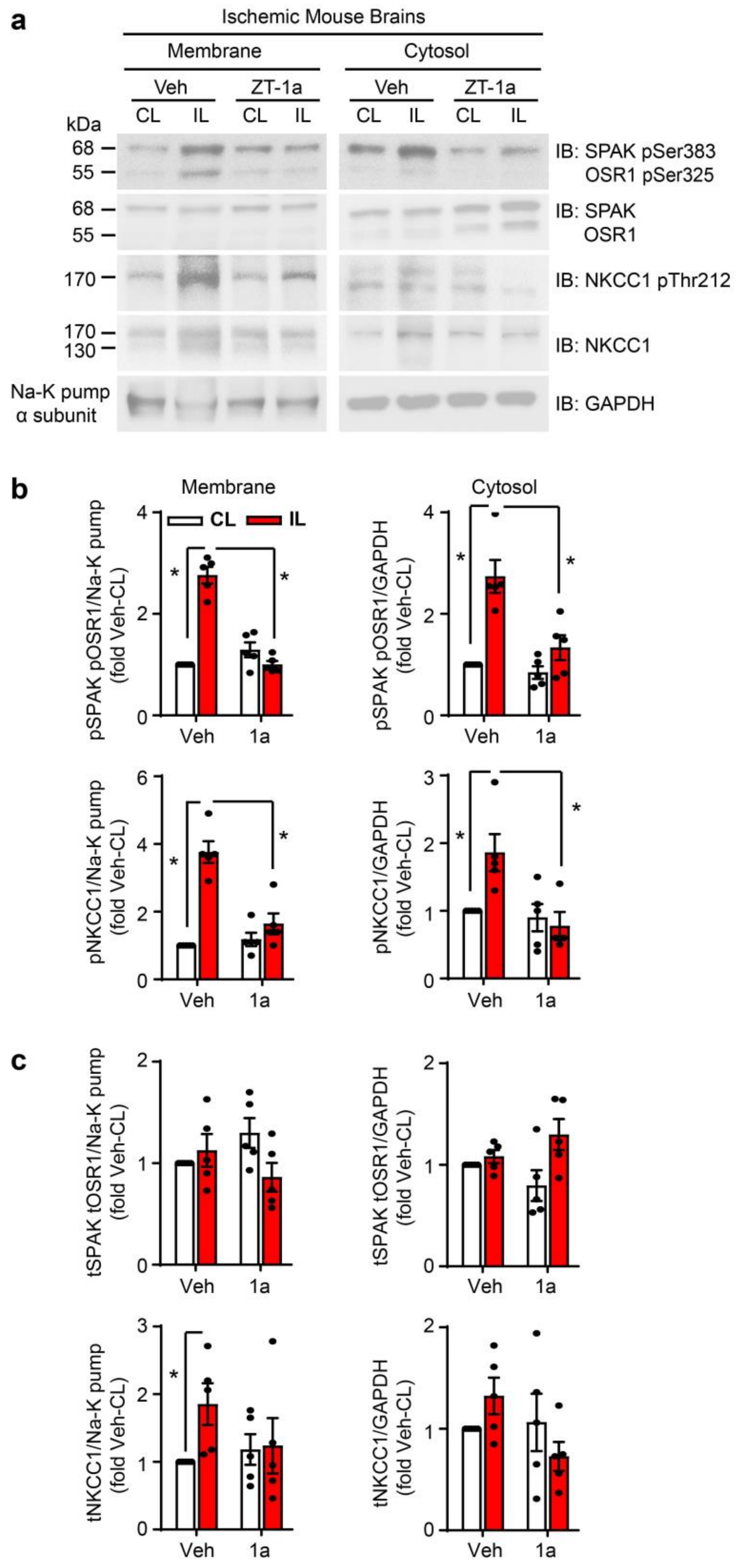

Supplemental Fig. 11 SPAK inhibitor ZT-1a blocks stroke-induced SPAK activation and NKCC1 protein phosphorylation in ischemic brains.

a Representative immunoblots (IB) of pSPAK/pOSR1 and pNKCC1 in mouse brains at 24 hrs post-reperfusion after ischemic stroke. Cytosol and crude membrane protein fractions were prepared from

contralateral (CL) and ipsilateral (IL) cerebral hemispheres. Vehicle (DMSO, 2 ml/kg) or ZT-1a (5.0 mg/kg) were administered with an initial half dose at 3 hr and the second half dose at 8 hr reperfusion. Na^{+}-K^{+} ATPase α-subunit and GAPDH served; as loading controls for membrane and cytosol fractions, respectively. **b-c** Summarized densitometric data from immunoblots (similar to those in panel **a**) of pSPAK/pOSR1, pNKCC1, tSPAK/tOSR1, and tNKCC1. Data are means ± SEM, n = 5 (male 3, female 2). * $p < 0.05$.

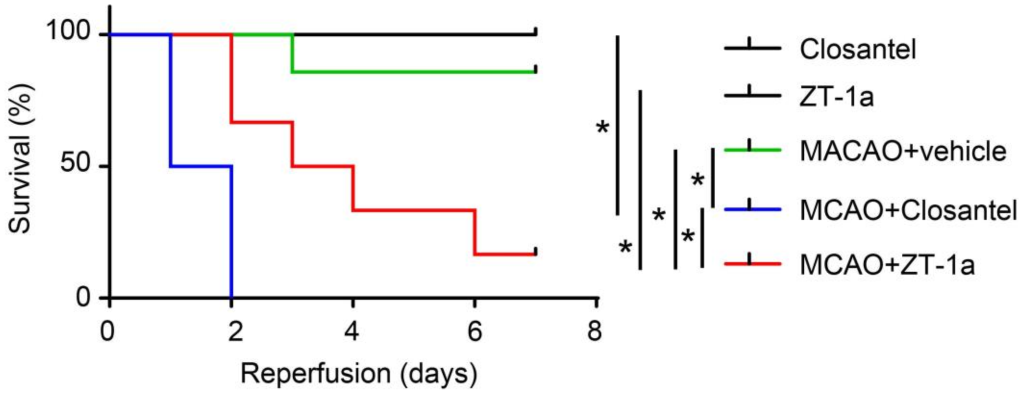

Supplemental Fig. 12 Kaplan-Meier survival curve of naïve and ischemic stroke mice treated with Closantel or ZT-1a.

Normal naïve C57BL/6J mice, or mice after tMCAO randomly received Closantel or ZT-1a treatment (25 mg/kg, i.p.) with an initial half dose at 3 hr and the second half dose at 8 hr reperfusion. Survival was monitored for 0-7 days post-treatment. No mortality was detected in the naïve mice treated with Closantel or ZT-1a. However, all ischemic stroke mice treated with Closantel died within 2 days, with a median survival of 1 day. In contrast, ZT-1a-treated stroke mice showed prolonged median survival (4 days). n = 3 per group (naïve+Closantel or naive+ZT-1a), n = 6 per group (tMCAO+vehicle or tMCAO+Closantel or tMCAO+ZT-1a), *$p < 0.05$, Log-rank test.

Supplementary Table 1. Cellular activity profile of inhibitors with the salicylic amide scaffold.

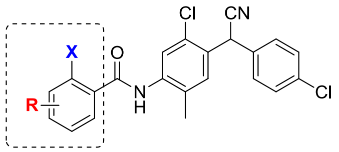

**Compound ID**	**Substituted benzoic acid unit**	**SPAK pSer373 (µM) Iso, hypo**	**NKCC1 pThr203 (µM) Iso, hypo**	**KCC3 pThr991 (µM) Iso, hypo**	**KCC3 pThr1048 (µM) Iso, hypo**
**1a (ZT-1a)**	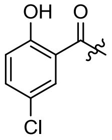	1, 3	1, 1	3, 3	1, 3
**1b**	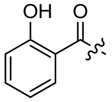	3, 3	3, 1	10, 10	3, 3
**1c**	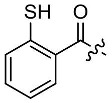	3, 3	1, 3	10, 10	3, 3
**1d**	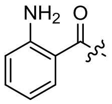	3, 3	1, 3	10, 10	3, 3
**1e**	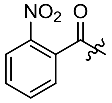	-	-	-	-
**1f**	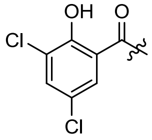	3, 3	1, 3	10, 10	3, 3
**1g**	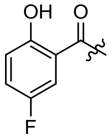	3, 3	1, 3	10, 10	3, 3
**1h**	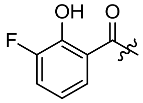	3, 3	1, 3	10, 10	3, 3
**1i**	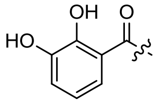	-	-	-	-
**1j**	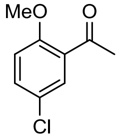	-	-	-	-

'-' showed no inhibition of phosphorylation of SPAK/NKCC1/KCC3, values are means ± SEM that represent 50% inhibition of SPAK pSer373 under Isotonic or Hypotonic conditions , n = 3.

Supplementary Table 2. ZT-1a kinase profiling data[6].

ZT-1a was tested at 10 and 1 μM against a panel of 140 recombinant kinases in an enzymatic activity-based assay performed by the International Centre for Kinase Profiling in the MRC Protein Phosphorylation and Ubiquitylation Unit. Values represent % residual kinase activity (mean ± SEM, n = 3). Kinases highlighted in Blue are inhibited ≥50% by ZT-1a.

**Kinases**	Concentration (μM)	
	10	1
ABL	68±16	85±4
AMPK (hum)	108±4	126±4
ASK1	100±11	101±3
Aurora A	65±1	73±19
Aurora B	92±15	119±6
BRK	103±2	116±12
BRSK1	88±22	108±14
BRSK2	104±0	115±14
BTK	73±2	113±1
CAMK1	71±31	60±5
CAMKKb	102±9	115±10
CDK2-Cyclin A	125±6	128±12
CDK9-Cyclin T1	108±1	104±0
CHK1	109±8	111±11
CHK2	68±6	106±1
CK1γ2	124±23	112±12
CK1δ	114±9	116±5
CK2	59±16	72±15
CLK2	33±45	68±12
CSK	106±29	96±8
DAPK1	159±11	131±8
DDR2	99±8	111±20
DYRK1A	103±1	119±7
DYRK2	97±10	111±15
DYRK3	56±15	113±6
EF2K	56±2	104±13

EIF2AK3	100±3	114±2
EPH-A2	78±11	89±23
EPH-A4	90±11	59±33
EPH-B1	80±19	77±15
EPH-B2	81±8	77±2
EPH-B3	84±6	88±6
EPH-B4	151±33	100±8
ERK1	109±18	107±16
ERK2	75±3	111±25
ERK5	114±5	120±9
ERK8	63±3	104±11
FGF-R1	101±15	93±6
GCK	97±3	112±4
GSK3b	38±12	39±4
HER4	84±1	85±8
HIPK1	90±4	98±13
HIPK2	91±4	90±14
HIPK3	76±2	84±3
IGF-1R	123±3	145±2
IKKb	78±3	101±0
IKKe	59±5	87±13
IR	86±13	105±2
IRAK1	95±7	128±2
IRAK4	127±11	127±0
IRR	95±4	105±5
JAK2	123±8	125±6
JNK1	110±5	106±4
JNK2	147±51	102±2
JNK3	60±10	63±15
Lck	127±14	112±13
LKB1	115±11	107±8
MAP4K3	88±2	134±5
MAP4K5	76±9	95±28
MAPKAP-K2	13±7	91±13
MAPKAP-K3	72±8	96±26

MARK1	91±5	104±16
MARK2	89±15	103±8
MARK3	68±8	71±6
MARK4	59±9	68±8
MEKK1	93±16	109±1
MELK	63±3	130±34
MINK1	108±6	107±5
MKK1	114±17	106±2
MKK2	81±27	126±2
MKK6	82±16	102±12
MLK1	81±21	94±4
MLK3	114±3	119±11
MNK1	87±2	78±19
MNK2	105±3	100±7
MPSK1	105±2	110±6
MSK1	112±14	114±6
MST2	113±2	115±4
MST3	113±15	109±8
MST4	63±0	63±10
NEK2a	91±6	85±12
NEK6	108±1	107±6
NUAK1	61±16	52±7
OSR1	98±3	106±9
p38a MAPK	113±2	128±7
p38b MAPK	124±0	124±1
p38d MAPK	112±2	95±1
p38g MAPK	100±6	100±11
PAK2	93±8	110±4
PAK4	51±3	81±16
PAK5	93±4	93±26
PAK6	75±4	69±2
PDGFRA	80±7	84±11
PDK1	69±17	92±6
PHK	107±2	97±3
PIM1	101±8	108±19

PIM2	93±3	93±5
PIM3	99±2	115±12
PINK	59±4	101±13
PKA	50±2	80±11
PKBa	91±4	104±1
PKBb	51±13	76±14
PKCa	83±2	97±4
PKCz	122±28	80±11
PKCγ	120±8	133±11
PKD1	79±10	89±0
PLK1	93±21	72±1
PRAK	41±7	78±13
PRK2	120±7	118±4
RIPK2	120±3	128±5
ROCK 2	101±10	109±6
RSK1	87±4	95±2
RSK2	101±3	116±5
S6K1	81±2	119±10
SGK1	58±5	73±14
SIK2	76±8	105±16
SIK3	88±0	104±9
SmMLCK	98±9	99±3
Src	84±13	107±4
SRPK1	73±20	104±5
STK33	100±5	114±7
SYK	93±1	93±12
TAK1	103±5	117±22
TAO1	104±2	66±2
TBK1	94±3	119±10
TESK1	77±10	109±3
TGFBR1	77±5	63±9
TIE2	73±11	139±74
TLK1	124±4	130±13
TrkA	91±7	108±9
TSSK1	74±6	99±2

TTBK1	102±2	119±5
TTBK2	98±34	101±32
TTK	91±5	104±2
ULK1	89±13	87±10
ULK2	49±9	110±11
VEG-FR	88±28	113±10
WNK1	122±35	125±17
YES1	98±7	109±3
ZAP70	97±9	120±1

*Results are presented as percentage of kinase activity in the presence of ZT-1a relative to that in its absence. Data are mean ± SEM. of triplicate determinations. Kinases showing with <50% remaining activity in the presence of 10 μM ZT-1a are highlighted in blue.

Supplemental Table 3. ZT-1a pharmacokinetic (PK) properties in naïve mice.

ZT-1a pharmacokinetics were determined following a single 5 mg/kg intravenous (IV) dose and a single 10 mg/kg oral (PO) dose in ICR mice (N = 3 at each time point). Blood samples were collected at 0.08, 0.25, 0.5, 1, 2, 4, 8, 10 & 24 hr (IV) & 0.25, 0.5, 1, 2, 4, 8, 10 & 24 hr (PO) post dose. Samples were subjected to drug assay by LC-MS/MS, and data were analyzed by WinNonlin V6.3. T_{max} = time of maximum plasma concentration, C_{max} = maximum plasma concentration, AUC = area under the curve (measure of exposure), $T_{1/2}$ = half life, CL = plasma clearance, Vz = volume of distribution, MRT, mean residence time; F = oral bioavailability.

		**Dose**	**$t_{1/2}$**	**T_{max}**	**C_{max}**	**C_0**	**$AUC_{(0-t)}$**	**$AUC_{(0-\infty)}$**	**Vz**	**Cl**	**$MRT_{(0-\infty)}$**	**F**
	**Route**	**(mg/kg)**	**hr**	**hr**	**ng/mL**	**ng/mL**	**ng*hr /mL**	**ng*hr /mL**	**L/kg**	**mL/kg*min**	**hr**	**%**
**Plasma**	IV	5	1.8	0.08	6910	14900	2340	2350	0.948	35.5	0.45	-
	PO	10	2.6	0.25	94	-	97.3	105	-	-	3.3	2.2

Reference:

1. Zhang J, Siew K, Macartney T, O'Shaughnessy KM, Alessi DR. Critical role of the SPAK protein kinase CCT domain in controlling blood pressure. *Human molecular genetics* **24**, 4545-4558 (2015).

2. Gasteiger E, Jung E, Bairoch A. SWISS-PROT: connecting biomolecular knowledge via a protein database. *Curr Issues Mol Biol* **3**, 47-55 (2001).

3. Kahle KT*, et al.* Peripheral motor neuropathy is associated with defective kinase regulation of the KCC3 cotransporter. *Science signaling* **9**, ra77 (2016).

4. Adragna NC*, et al.* Regulated phosphorylation of the K-Cl cotransporter KCC3 is a molecular switch of intracellular potassium content and cell volume homeostasis. *Frontiers in cellular neuroscience* **9**, 255 (2015).

5. Lenart B, Kintner DB, Shull GE, Sun D. Na-K-Cl cotransporter-mediated intracellular Na+ accumulation affects Ca2+ signaling in astrocytes in an in vitro ischemic model. *The Journal of neuroscience : the official journal of the Society for Neuroscience* **24**, 9585-9597 (2004).

6. Bain J*, et al.* The selectivity of protein kinase inhibitors: a further update. *The Biochemical journal* **408**, 297-315 (2007).
