# Supplementary material for: Modulation of brain cation-Cl− cotransport via the SPAK kinase inhibitor ZT-1a
Source: Nat Commun. 2020 Jan 7;11:78. doi: 10.1038/s41467-019-13851-6 (PMC6946680; doi:10.1038/s41467-019-13851-6)
Supplement: Supplementary file 3 — Reporting Summary [file 41467_2019_13851_MOESM3_ESM.pdf]

nature research

Corresponding author(s): NCOMMS-18-34527

Last updated by author(s): Nov 19, 2019

# Reporting Summary

Nature Research wishes to improve the reproducibility of the work that we publish. This form provides structure for consistency and transparency in reporting. For further information on Nature Research policies, seeAuthors & Referees and theEditorial Policy Checklist .

## Statistics

For all statistical analyses, confirm that the following items are present in the figure legend, table legend, main text, or Methods section.

| n/a | Confirmed | |
|---|---|---|
| ☐ | ☒ | The exact sample size (*n*) for each experimental group/condition, given as a discrete number and unit of measurement |
| ☐ | ☒ | A statement on whether measurements were taken from distinct samples or whether the same sample was measured repeatedly |
| ☐ | ☒ | The statistical test(s) used AND whether they are one- or two-sided<br>*Only common tests should be described solely by name; describe more complex techniques in the Methods section.* |
| ☒ | ☐ | A description of all covariates tested |
| ☒ | ☐ | A description of any assumptions or corrections, such as tests of normality and adjustment for multiple comparisons |
| ☐ | ☒ | A full description of the statistical parameters including central tendency (e.g. means) or other basic estimates (e.g. regression coefficient) AND variation (e.g. standard deviation) or associated estimates of uncertainty (e.g. confidence intervals) |
| ☐ | ☒ | For null hypothesis testing, the test statistic (e.g. *F*, *t*, *r*) with confidence intervals, effect sizes, degrees of freedom and *P* value noted<br>*Give P values as exact values whenever suitable.* |
| ☒ | ☐ | For Bayesian analysis, information on the choice of priors and Markov chain Monte Carlo settings |
| ☒ | ☐ | For hierarchical and complex designs, identification of the appropriate level for tests and full reporting of outcomes |
| ☒ | ☐ | Estimates of effect sizes (e.g. Cohen's *d*, Pearson's *r*), indicating how they were calculated |

*Our web collection on statistics for biologists contains articles on many of the points above.*

## Software and code

Policy information about availability of computer code

| | |
|---|---|
| Data collection | A description of the software and code has been included in the Methods. |
| Data analysis | A description of the software and code has been included in the Methods. |

For manuscripts utilizing custom algorithms or software that are central to the research but not yet described in published literature, software must be made available to editors/reviewers. We strongly encourage code deposition in a community repository (e.g. GitHub). See the Nature Research guidelines for submitting code & software for further information.

## Data

Policy information about availability of data

All manuscripts must include a data availability statement. This statement should provide the following information, where applicable:

- Accession codes, unique identifiers, or web links for publicly available datasets
- A list of figures that have associated raw data
- A description of any restrictions on data availability

*Provide your data availability statement here.*

# Field-specific reporting

Please select the one below that is the best fit for your research. If you are not sure, read the appropriate sections before making your selection.

☒ Life sciences ☐ Behavioural & social sciences ☐ Ecological, evolutionary & environmental sciences

For a reference copy of the document with all sections, see nature.com/documents/nr-reporting-summary-flat.pdf

# Life sciences study design

All studies must disclose on these points even when the disclosure is negative.

| | |
|---|---|
| Sample size | For all experiments at least three independent replicates were taken for each data point, unless otherwise indicated. The number of animals studied was 80% powered to detect 20% changes with α (2-sided) = 0.05. |
| Data exclusions | No data were excluded from the datasets. |
| Replication | Two independent experiments at least were repeated with similar results, the number of replicates are mentioned in the figure legends and/or the methods section. |
| Randomization | Animals were assigned randomly to experimental and control groups, and within animal controls were performed wherever possible. Mice analyzed were litter mates and sex-matched whenever possible. |
| Blinding | During the in vivo mouse/rat experiments the investigators were unaware of the outcome of the experiments. So, for data collection, the investigators were totally blind towards the data sampling.<br>For example, administration of compounds was carried out as a blinded experiment (all information about the expected outputs and the nature of used compounds were kept from the animal-technicians). |

# Reporting for specific materials, systems and methods

We require information from authors about some types of materials, experimental systems and methods used in many studies. Here, indicate whether each material, system or method listed is relevant to your study. If you are not sure if a list item applies to your research, read the appropriate section before selecting a response.

## Materials & experimental systems

| n/a | Involved in the study | |
|---|---|---|
| ☐ | ☒ | Antibodies |
| ☐ | ☒ | Eukaryotic cell lines |
| ☒ | ☐ | Palaeontology |
| ☐ | ☒ | Animals and other organisms |
| ☒ | ☐ | Human research participants |
| ☒ | ☐ | Clinical data |

## Methods

| n/a | Involved in the study | |
|---|---|---|
| ☒ | ☐ | ChIP-seq |
| ☒ | ☐ | Flow cytometry |
| ☐ | ☒ | MRI-based neuroimaging |

## Antibodies

| | |
|---|---|
| Antibodies used | The Division of Signal Transduction Therapy Unit at the University of Dundee (RRID:SCR_011633) supplied antibodies to KCC3 (1 µg/ml, S701C), KCC3A phosphoT991 (1 µg/ml, S959C), KCC3A phosphoT1048 (1 µg/ml, S961C), NKCC1 (1 µg/ml, S022D), NKCC1 phospho-T203/T207/T212 (1 µg/ml, S763B), WNK1 (1 µg/ml, S079B), SPAK (1 µg/ml, S551D), OSR1 (1 µg/ml, S850C), SPAK/OSR1 (S-motif) phosphoS373/S325 (1 µg/ml, S670B) and full-length human ERK1(1 µg/ml, S221B). The pan-KCC2 antibody (NeuroMab clone N1/12) was from NeuroMab. The FLAG M2 antibody (1 µg/ml, F3165) was from Sigma. The KCC4 antibody was from Novus Biologicals (0.4 µg/ml, NBP1-85133). Antibodies to GSK-3β phophoS9 (1:1000 dilution, #9336), GAPDH (0.1 µg/ml, #2118) and β-Actin (1:5000 dilution, 8H10D10) were from Cell Signaling Technology. Anti-pGSK-3α/β (1:1000 dilution, #OPA1-03083) was from Thermo Scientific. Anti-Na+-K+-ATPase alpha subunit (1:1000 dilution, #a5c) was from the Developmental Studies Hybridoma Bank (RRID:SCR_013527, Iowa City, IA). Horseradish peroxidase-coupled secondary antibodies for immunoblotting were from Pierce. IgG used in control immunoprecipitation experiments was affinity-purified from pre-immune sera using Protein G-Sepharose. |
| Validation | Both primary and secondary antibodies are used on a weekly basis in our lab. Additional information on the antibodies are available on the manufacture's websites. |

## Eukaryotic cell lines

Policy information about cell lines

| | |
|---|---|
| Cell line source(s) | HEK293 cell line was purchased from Sigma (Now is Merck, cat. no. 85120602-1VL) https://www.sigmaaldrich.com/catalog/product/sigma/cb_85120602?lang=en&region=GB&gclid=Cj0KCQiA3IPgBRCAARIsABb-iGJxDYqU69NrdhoA9MwXMDjVqrYgt8tD_eHfvcy7t-aMGGLZA_PlrzQaAonzEALw_wcB More information in Methods section. |
| Authentication | Cells that were purchased from Sigma (Now is Merck) were authenticated by the Sigma (Now is Merck)using short, tandem-repeat profiling. |

| | |
|---|---|
| Mycoplasma contamination | Mycoplasma tests were performed monthly using LookOut® Mycoplasma PCR Detection Kit Sigma (Now is Merck). No infection has been detected during experiments presented in this study. |
| Commonly misidentified lines (See ICLAC register) | No cell lines used in this study misidentified cell lines. |

## Animals and other organisms

Policy information about studies involving animals; ARRIVE guidelines recommended for reporting animal research

| | |
|---|---|
| Laboratory animals | Mouse, C57BL/6J, both male and female, 9-14 weeks of age |
| Wild animals | The study did not involve wild animals. |
| Field-collected samples | The study did not involve samples collected from the field. |
| Ethics oversight | The University of Pittsburgh Institutional Animal Care and Use Committee |

Note that full information on the approval of the study protocol must also be provided in the manuscript.

## Magnetic resonance imaging

### Experimental design

| | |
|---|---|
| Design type | Description of research mice or rat used for experiments can be found in the relevant figure legends and Methods. |
| Design specifications | N/A |
| Behavioral performance measures | N/A |

### Acquisition

| | |
|---|---|
| Imaging type(s) | Structural, Diffusion Tensor |
| Field strength | 11.7T |
| Sequence & imaging parameters | T2 - MSME, TE/TR = 80/3000 ms, NA = 16, 15 slices, slice thickness = 0.5 mm, Axial orientation, 160 x 160 matrix, 16 x 16 mm FOV.<br>DTI - Spin Echo, TE/TR = 22/2800 ms, NA = 2, 25 slices, slice thickness = 0.5 mm, Axial orientation, 160 x 160 matrix, 16 x 16 mm FOV, 30 directions, 5 A0 images, δ/Δ = 5/11 ms, b = 3000 s/mm2 |
| Area of acquisition | Whole brain |

Diffusion MRI ☒ Used ☐ Not used

Parameters: 30 directions, 5 A0 images, δ/Δ = 5/11 ms, b = 3000 s/mm2, single shell

### Preprocessing

| | |
|---|---|
| Preprocessing software | DSI Studio |
| Normalization | Data were not normalized |
| Normalization template | N/A |
| Noise and artifact removal | N/A |
| Volume censoring | N/A |

### Statistical modeling & inference

| | |
|---|---|
| Model type and settings | N/A |
| Effect(s) tested | N/A |

Specify type of analysis: ☐ Whole brain ☒ ROI-based ☐ Both

Anatomical location(s): *Describe how anatomical locations were determined (e.g. specify whether automated labeling algorithms or probabilistic atlases were used).*

| | |
|---|---|
| Statistic type for inference (See Eklund et al. 2016) | N/A |
| Correction | N/A |

## Models & analysis

| n/a | Involved in the study |
|---|---|
| ☒ | ☐ Functional and/or effective connectivity |
| ☒ | ☐ Graph analysis |
| ☒ | ☐ Multivariate modeling or predictive analysis |
