# Supplementary figures and images for: Modulation of brain cation-Cl− cotransport via the SPAK kinase inhibitor ZT-1a
Source: Nat Commun. 2020 Jan 7;11:78. doi: 10.1038/s41467-019-13851-6 (PMC6946680; doi:10.1038/s41467-019-13851-6)

**Fig. 1**

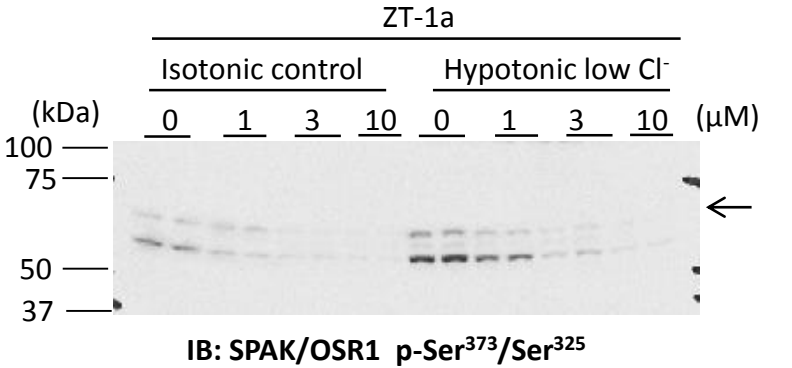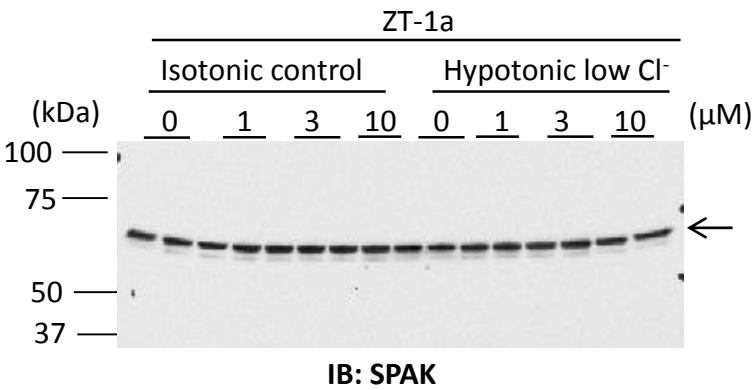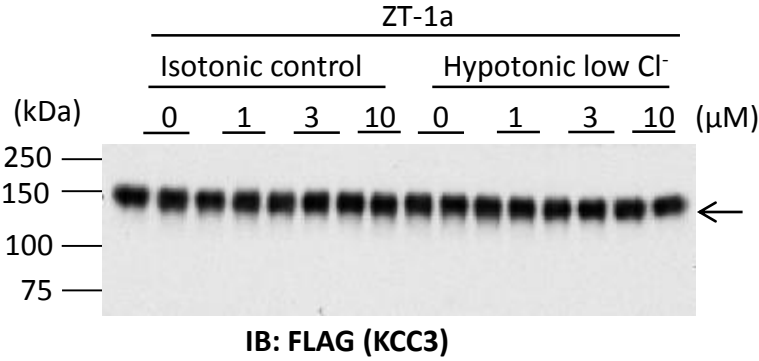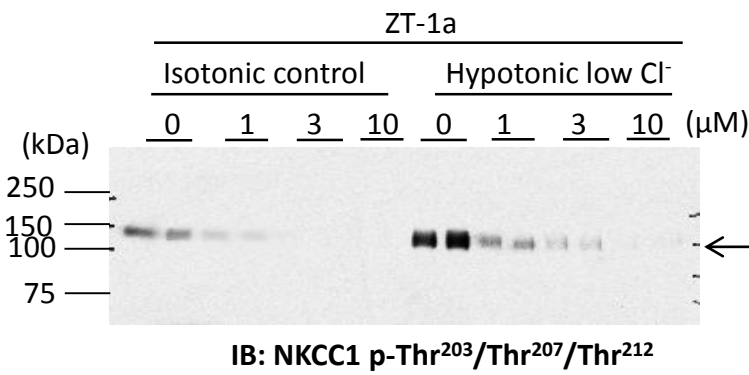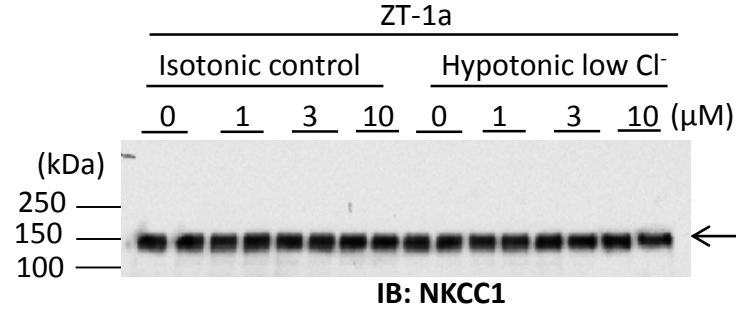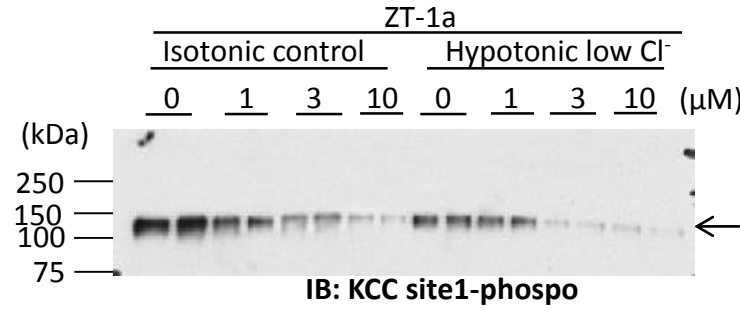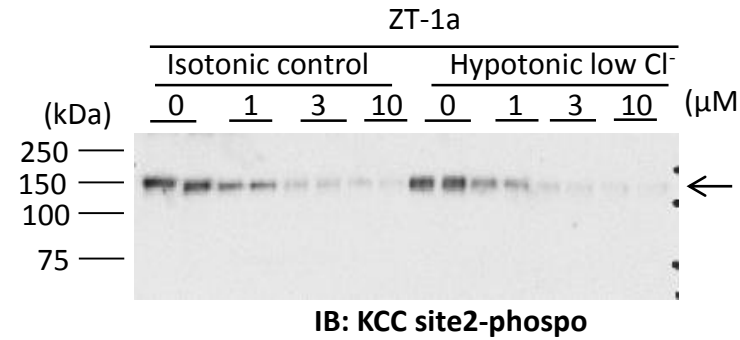

**Fig. 1**

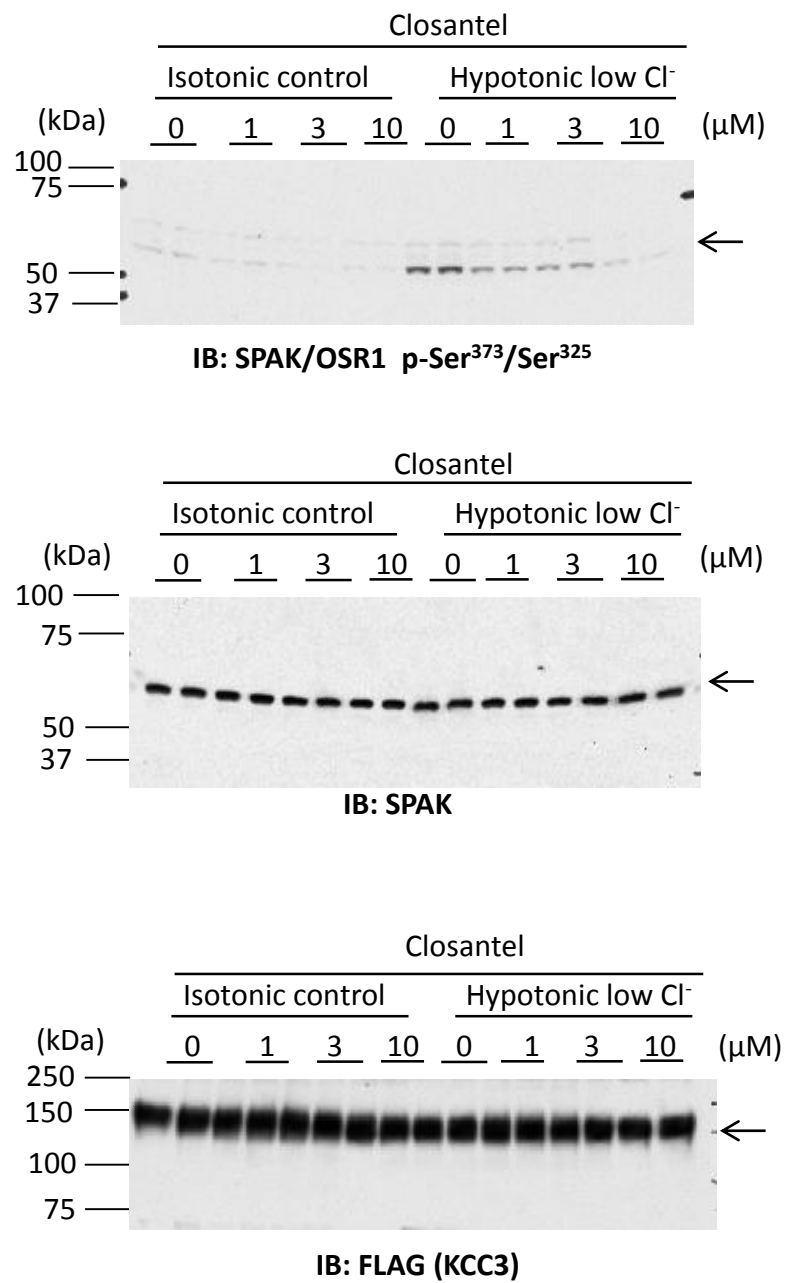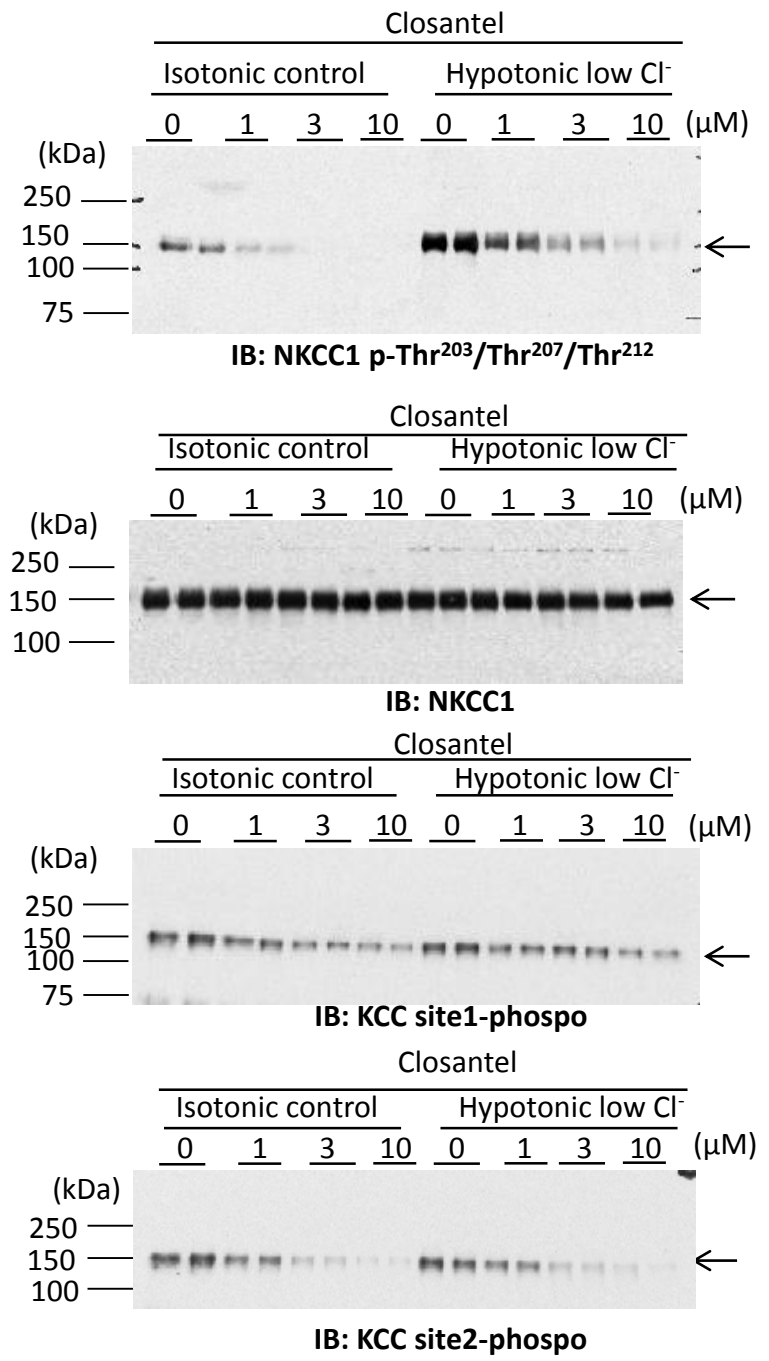

**Fig. 1**

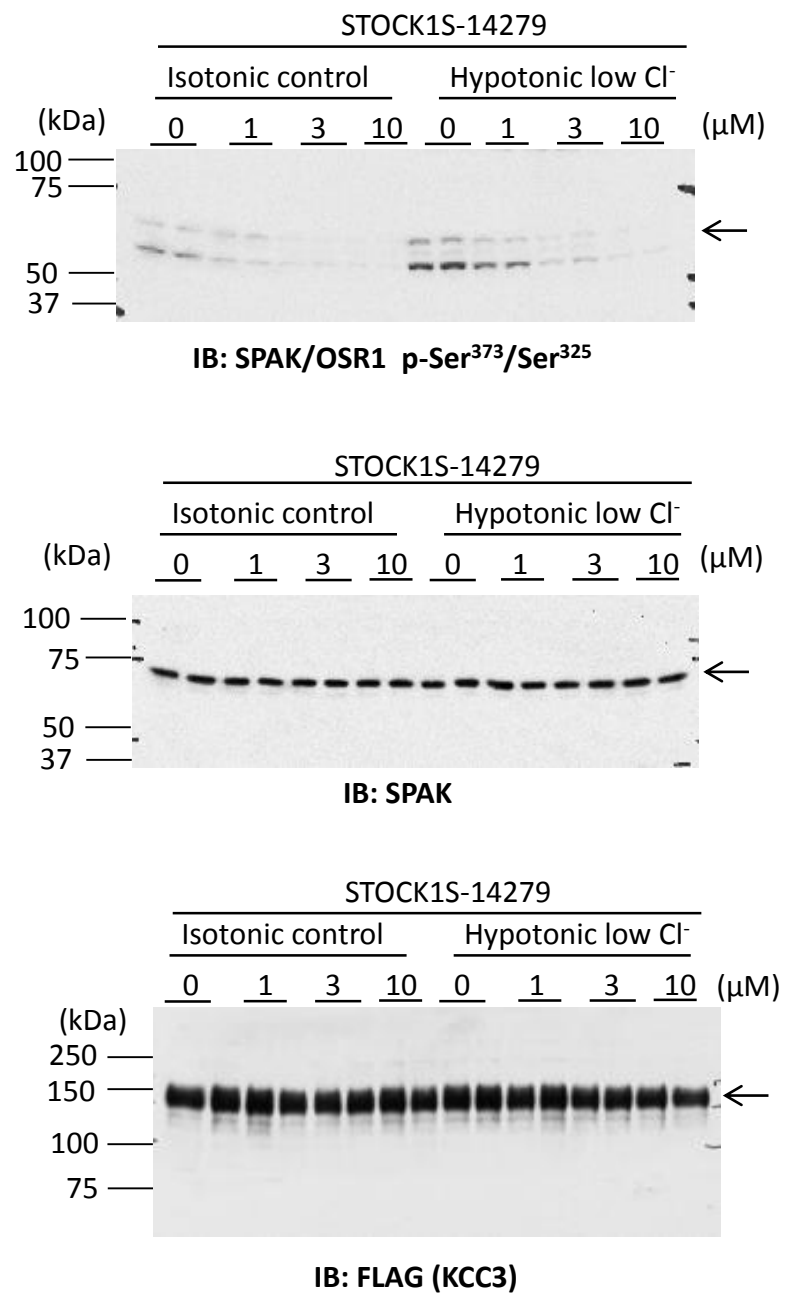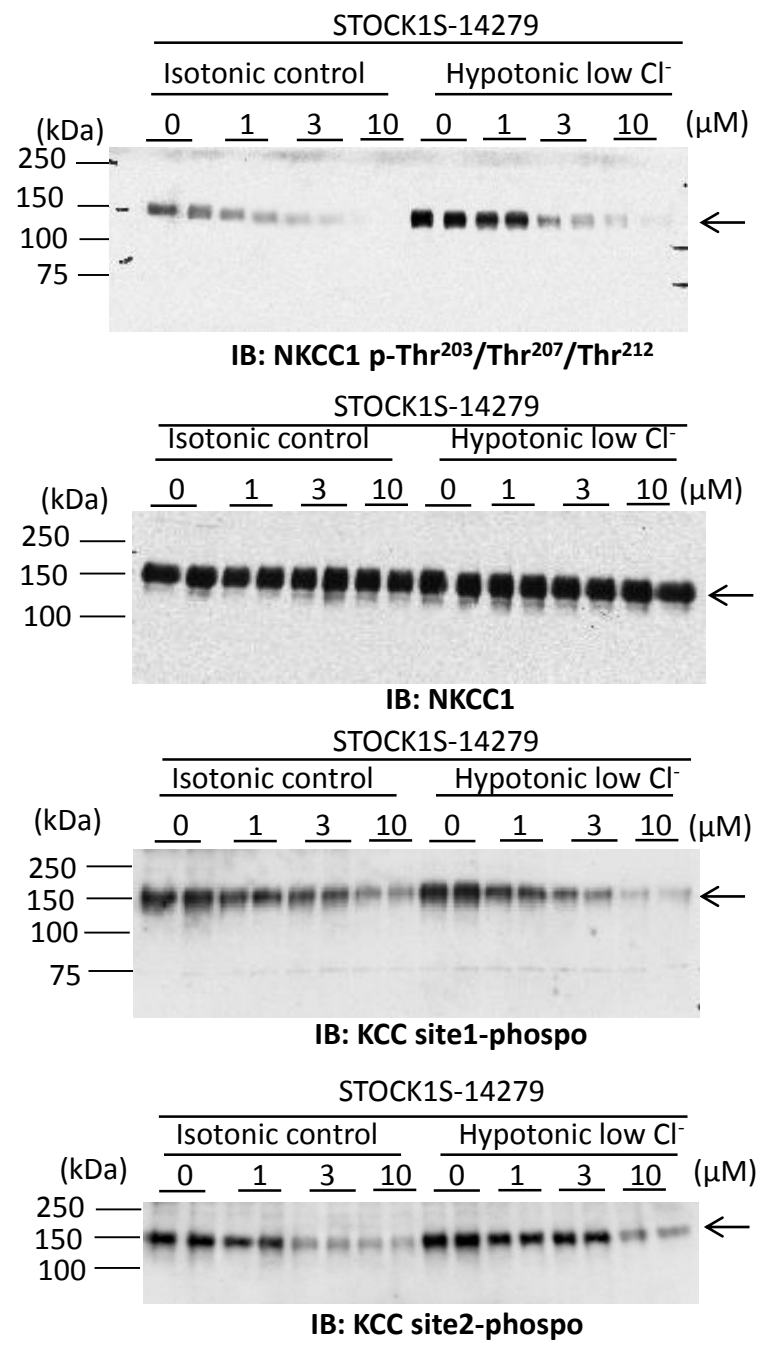

**Fig. 1**

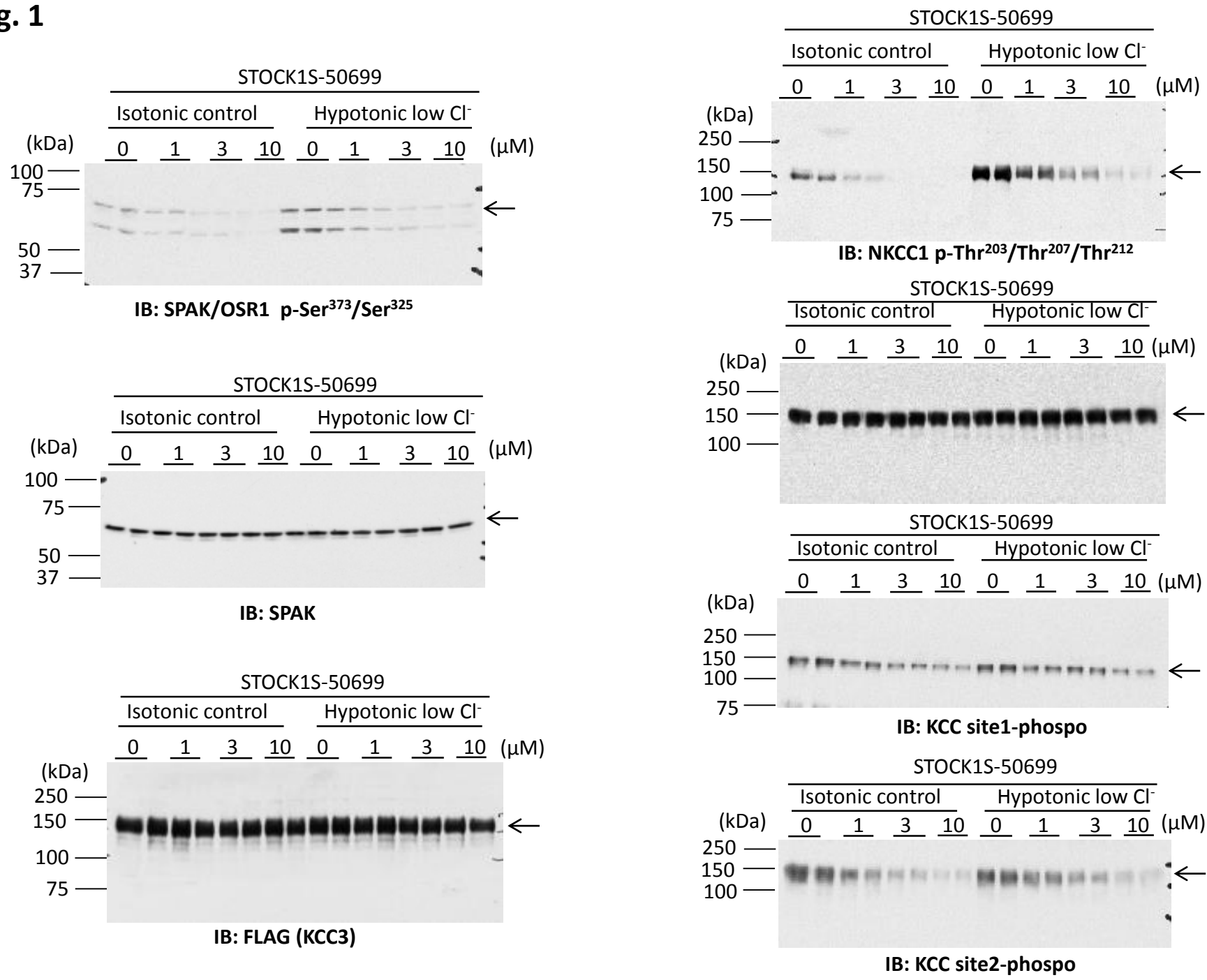

**Fig. 4**

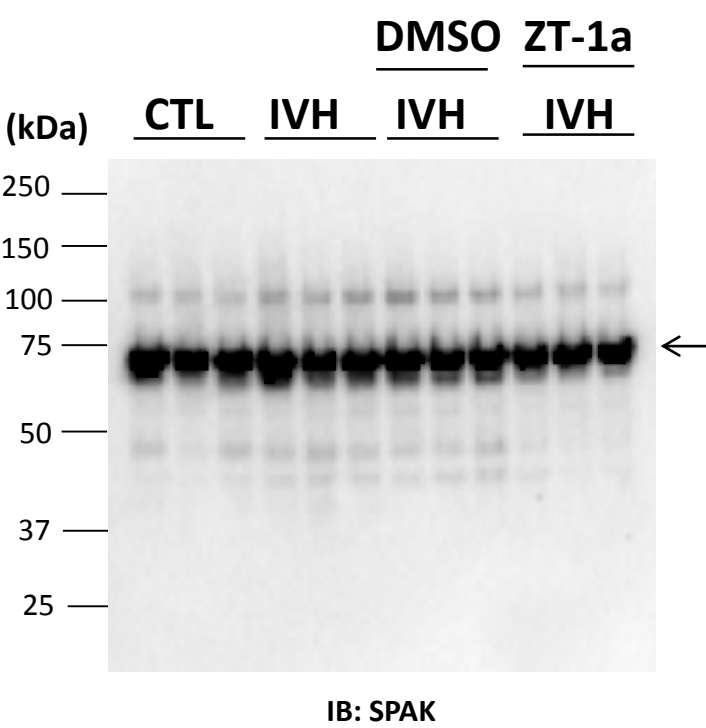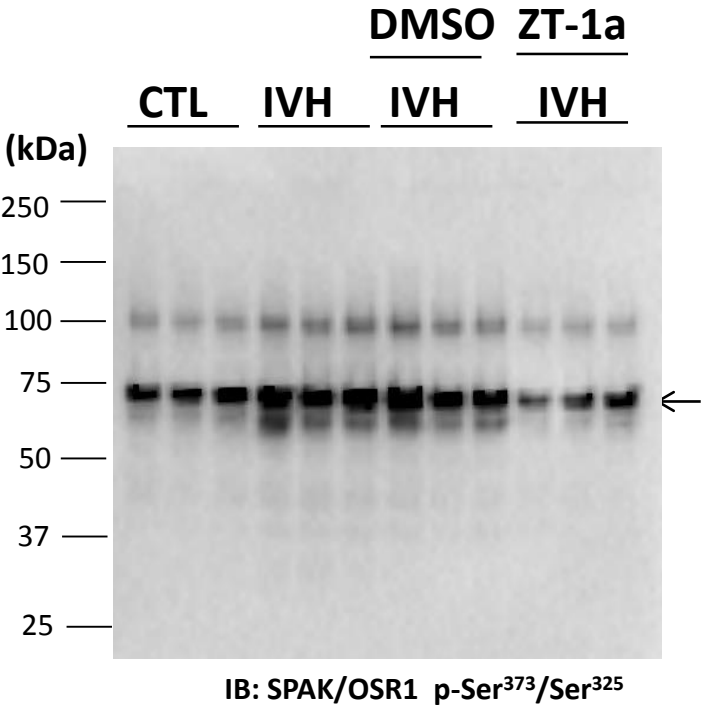

**Fig. 4**

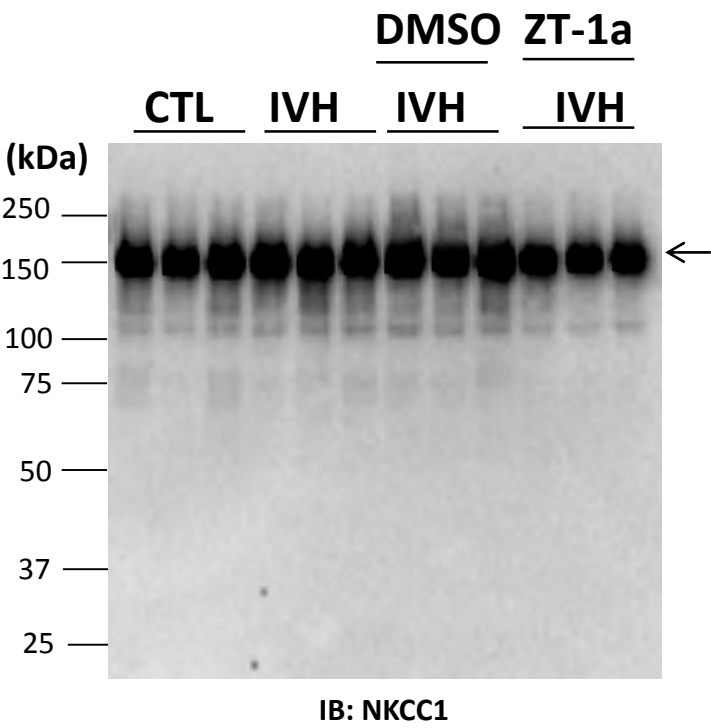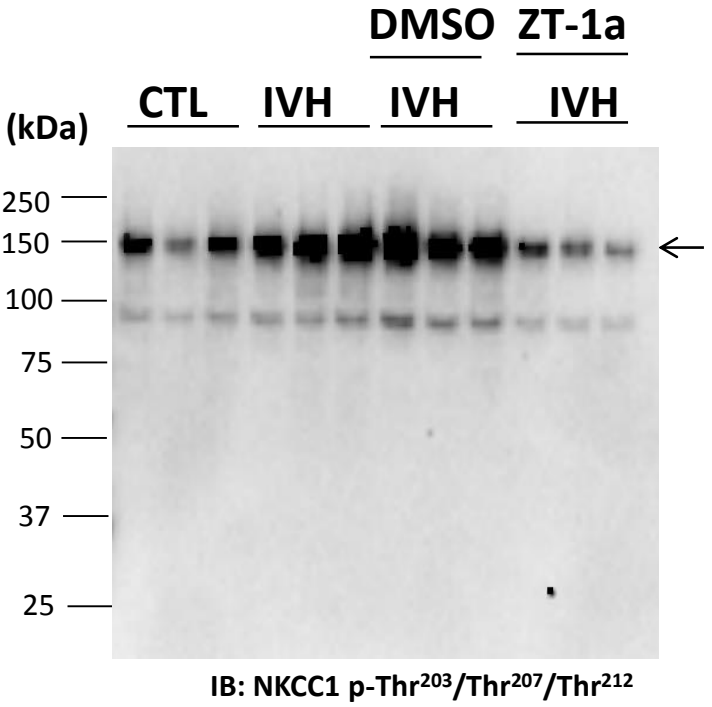

**Fig. 4**

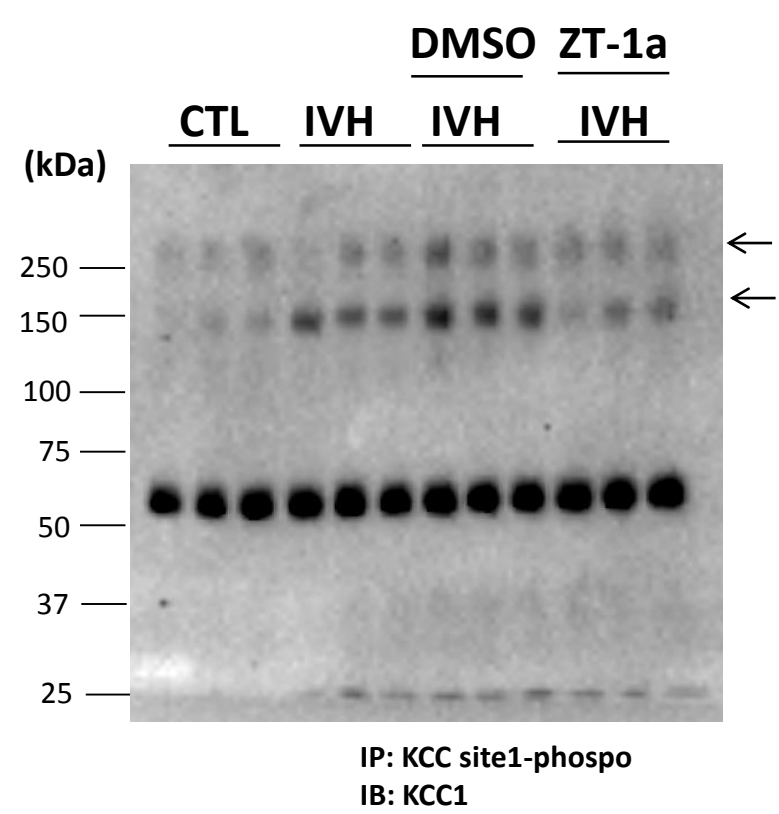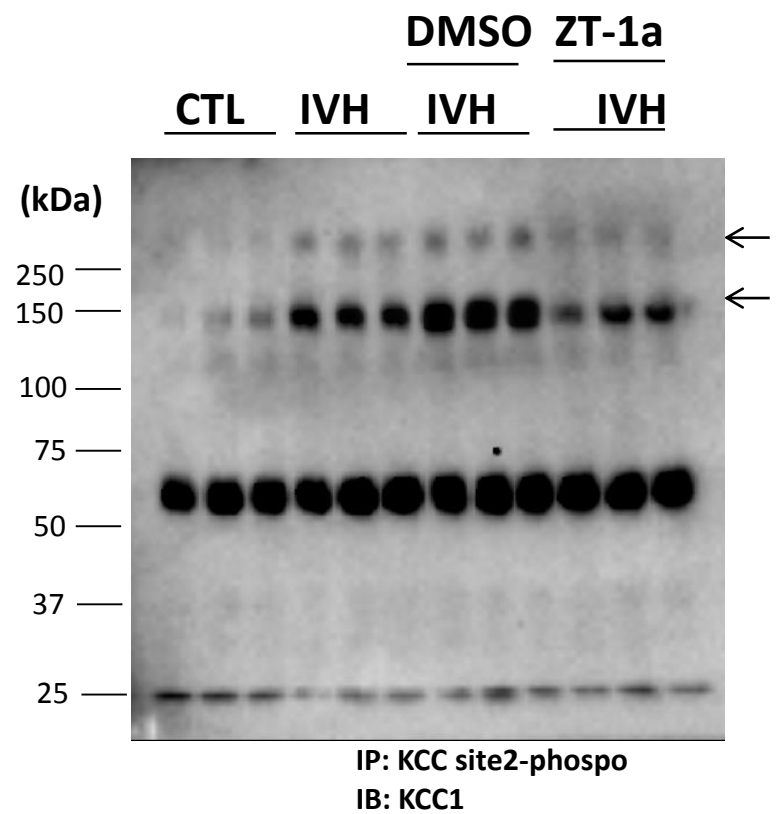

**Fig. 4**

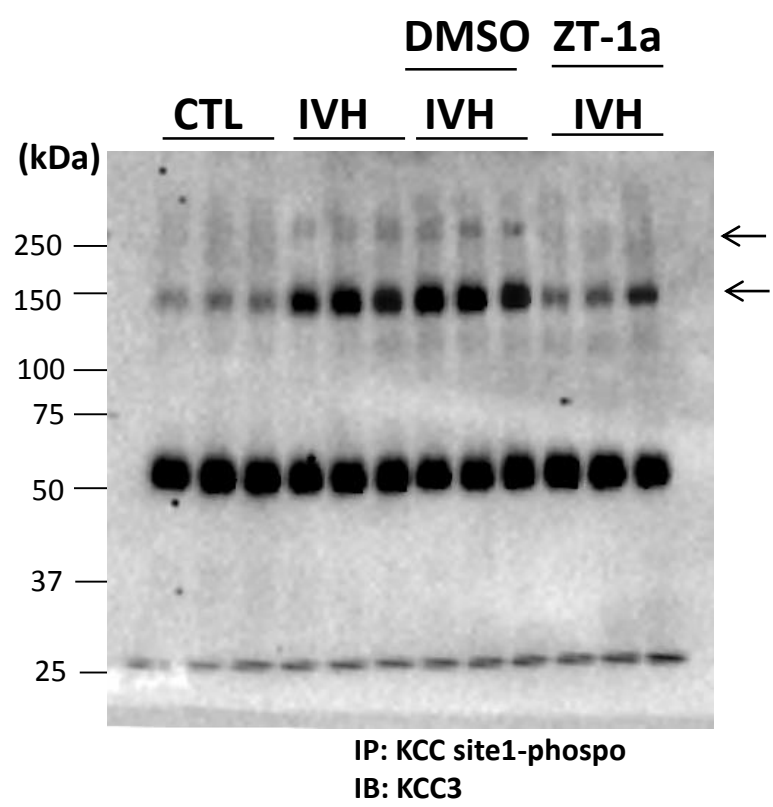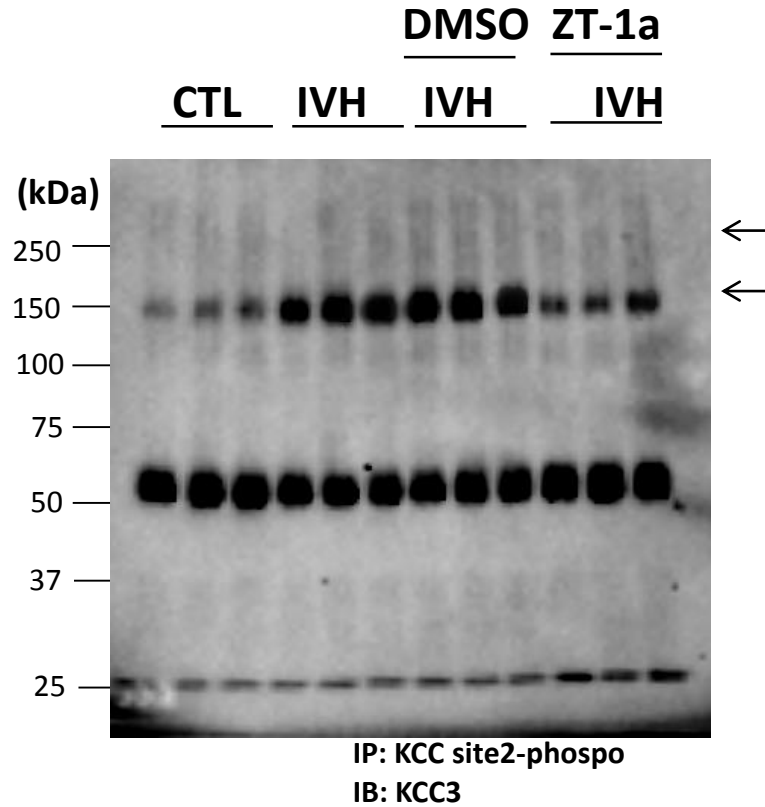

**Fig. 4**

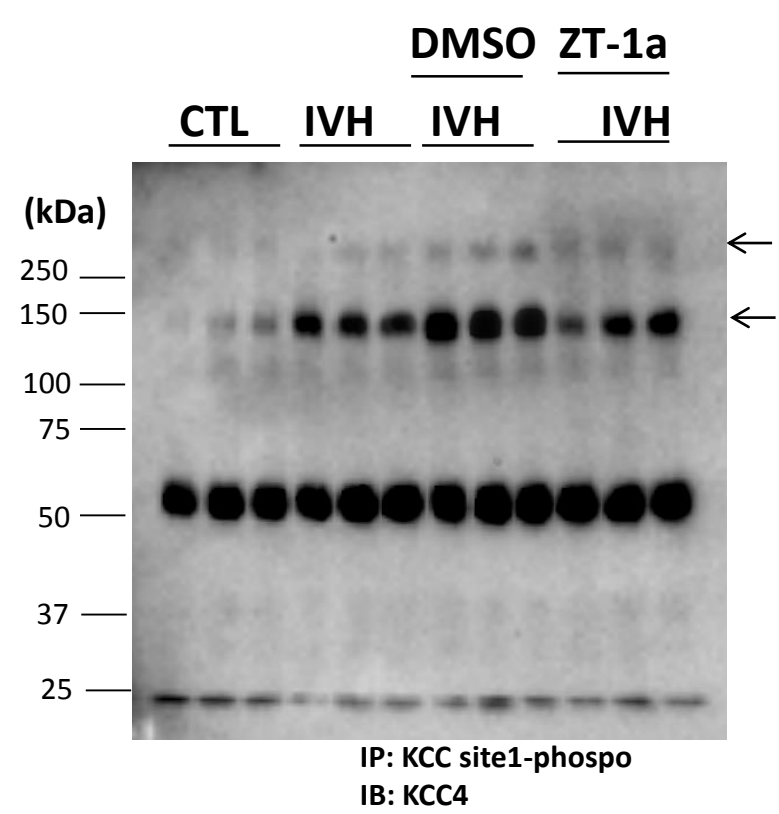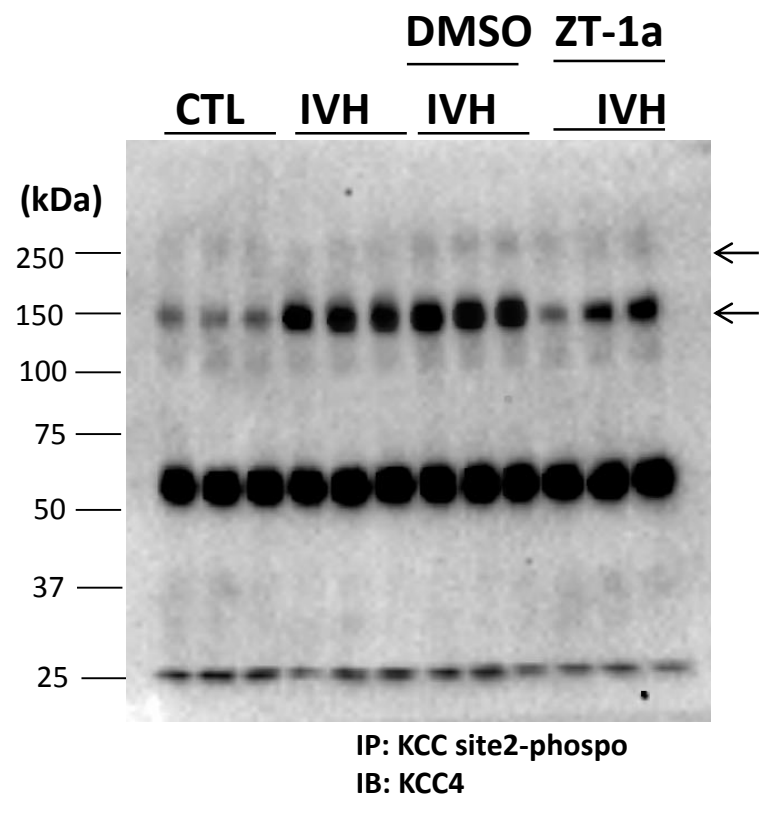

**Fig. 4**

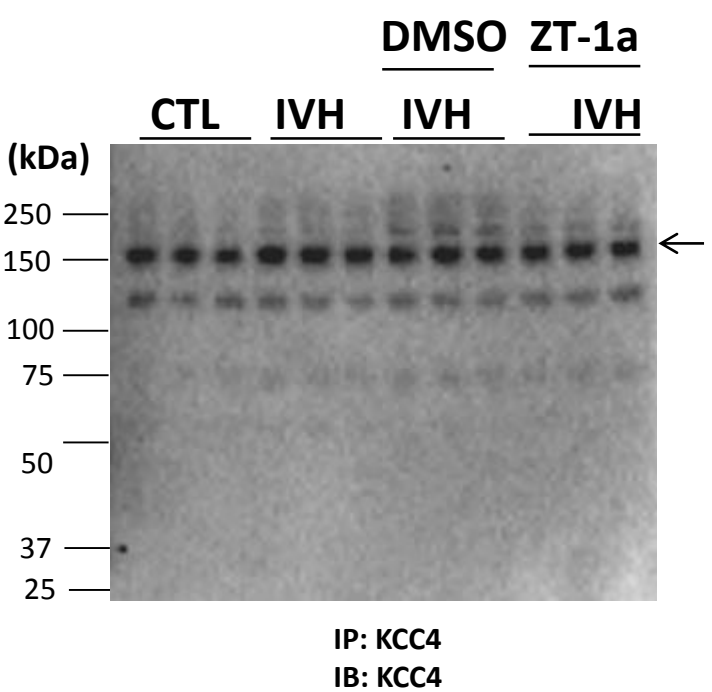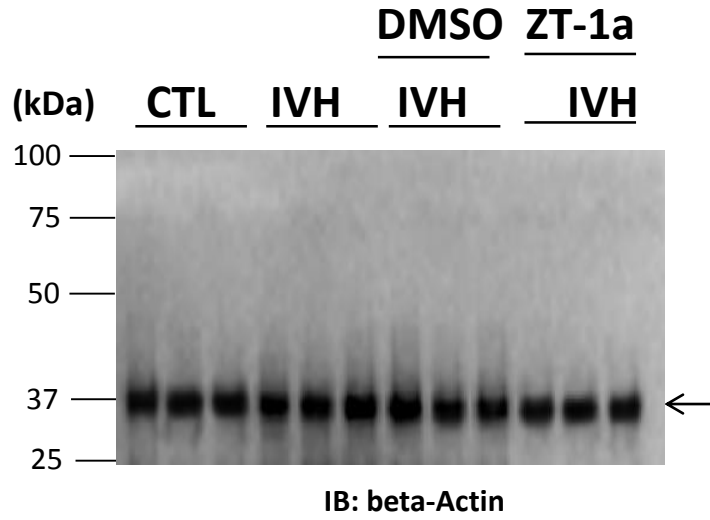

**Fig. 8**

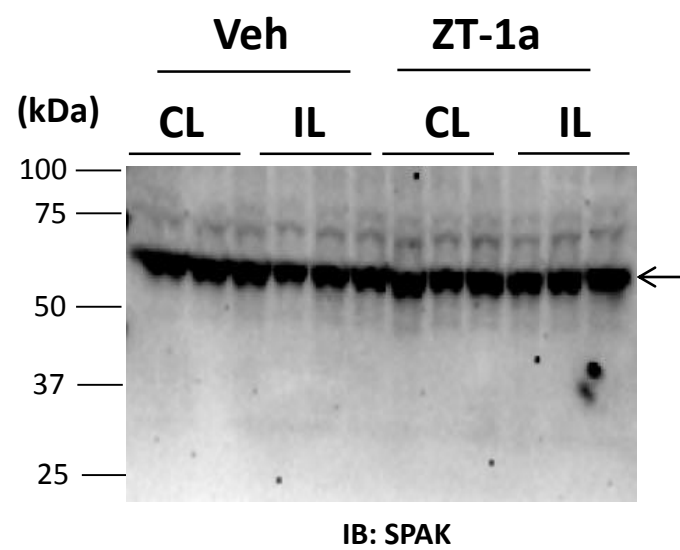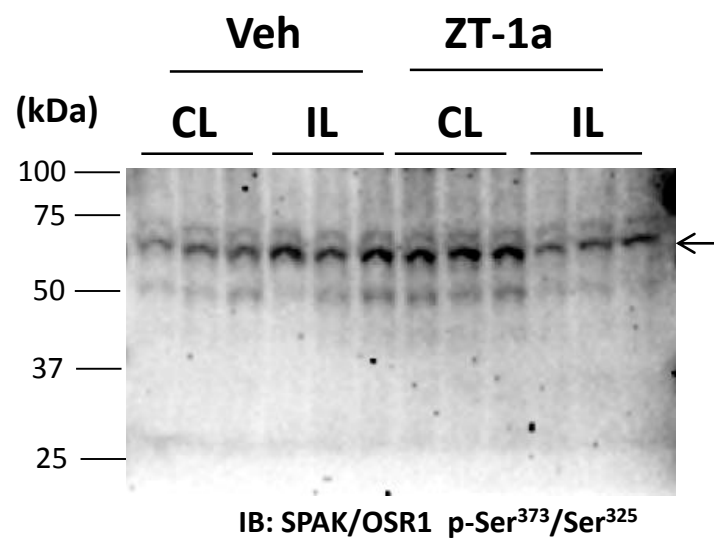

**Fig. 8**

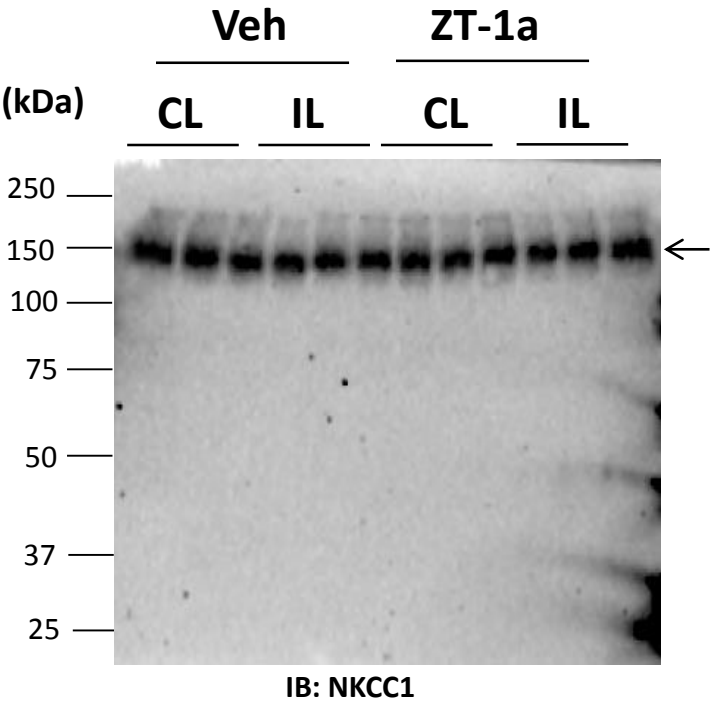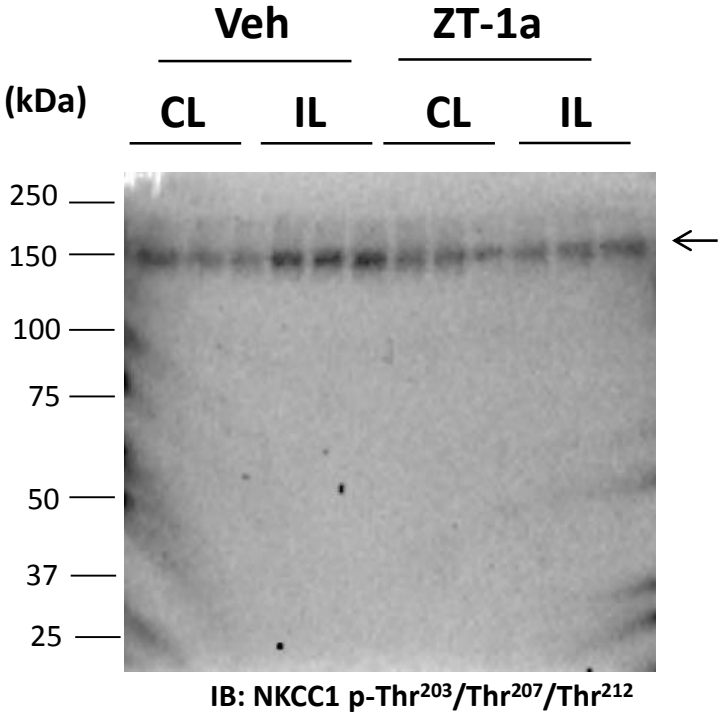

**Fig. 8**

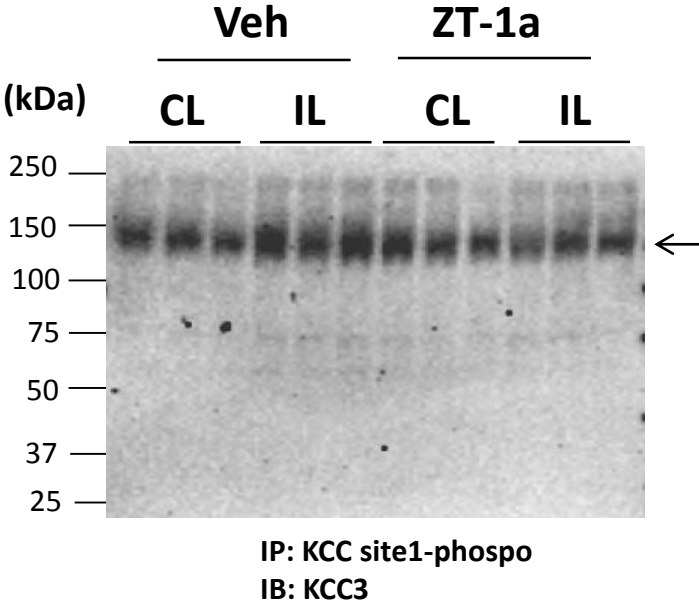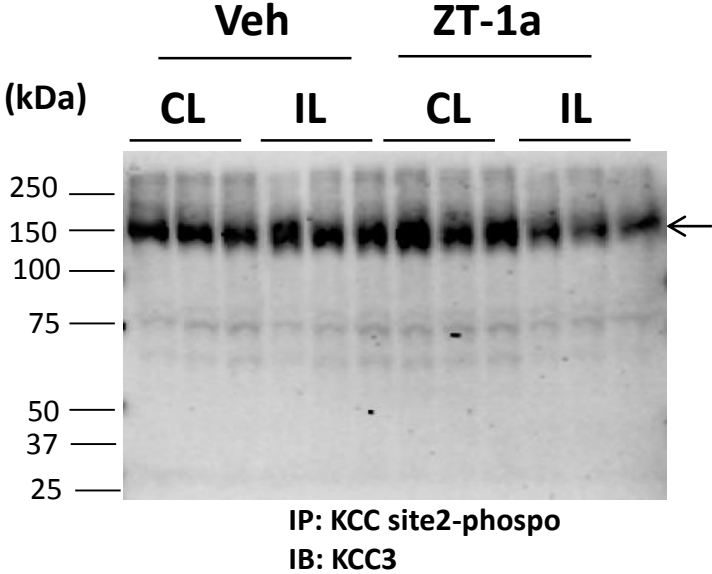

**Fig. 8**

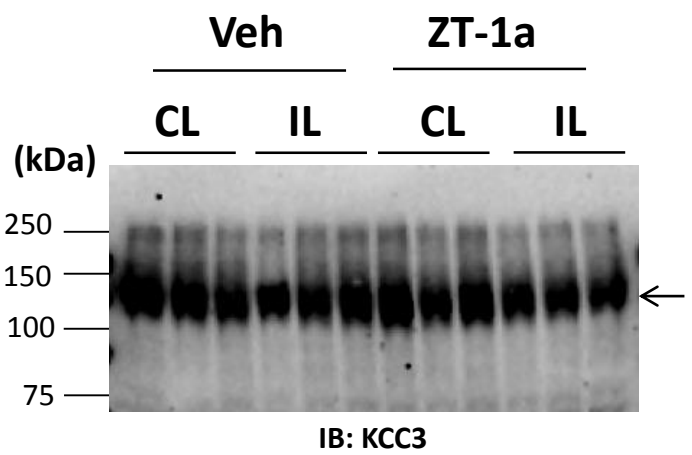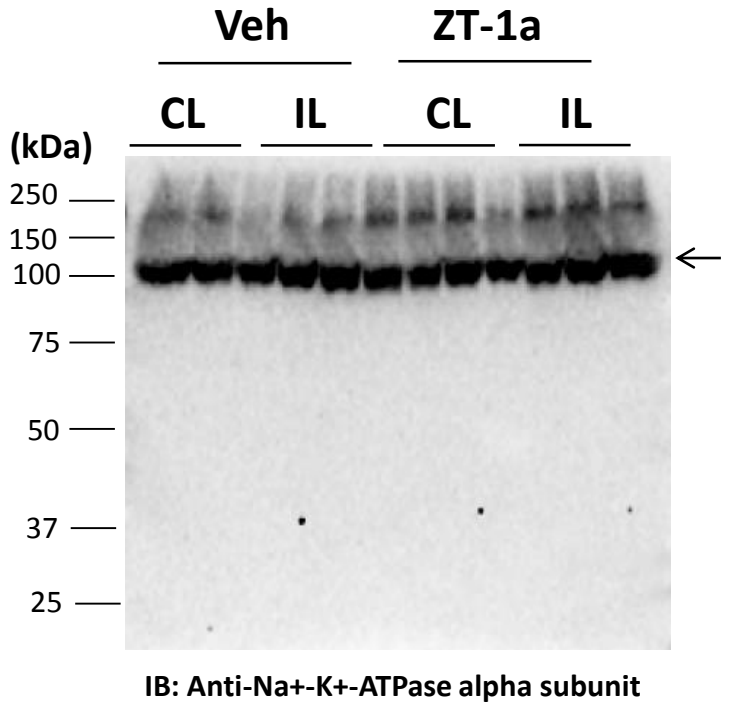

Supplement: Supplementary file 4 — Source Data [file 41467_2019_13851_MOESM4_ESM.zip › Source Data - Supplementary Western Blots.pdf]
